# Humanistic Therapy for Young People: Client-Perceived Helpful Aspects, Hindering Aspects, and Processes of Change

**DOI:** 10.1007/s10826-024-02955-3

**Published:** 2025-01-14

**Authors:** Mick Cooper, Stephanie Smith, Amy Louise Sumner, Jon Eilenberg, Jasmine Childs-Fegredo, Siobhan Kelly, Praveen Subramanian, Joanna Holmes, Michael Barkham, Peter Bower, Karen Cromarty, Charlie Duncan, Susan Hughes, Peter Pearce, Tiffany Rameswari, Gemma Ryan, David Saxon, Megan Rose Stafford

**Affiliations:** 1 School of Psychology, University of Roehampton, London, UK; 2 Research and Policy, National Children’s Bureau, London, UK; 3 Policy Department, British Association for Counselling and Psychotherapy, Leicestershire, UK; 4 Clinical and Applied Psychology Unit, Department of Psychology, University of Sheffield, Sheffield, UK; 5 NIHR School for Primary Care Research, Centre for Primary Care and Health Services Research, University of Manchester, Manchester, UK; 6 Independent Consultant, Durham, UK; 7 Research Department, British Association for Counselling and Psychotherapy, Leicestershire, UK; 8 Faculty of Applied Social & Organisational Sciences, Metanoia Institute, London, UK; 9 School of Psychology, University of Sheffield, Sheffield, UK; 10 Present Address: Centre for Evidence and Implementation, London, UK; 11 Present Address: Department of Psychology, University of Westminster, London, UK; 12 Present Address: Tænketanken DEA, Copenhagen, Denmark; 13 Present Address: School of Education, Language and Psychology, York St John University, York, UK; 14 Present Address: Enfield Therapy Service, Enfield & Haringey Mental Health NHS Trust, London, UK

**Keywords:** Young people, Humanistic psychotherapy, Person-centered therapy, Process-outcome research, Thematic analysis

## Abstract

This qualitative study aimed to establish aspects of humanistic therapy that young people (13–16 years old) perceived as helpful and hindering, and to test a novel method for identifying perceived processes of change. A “medium q” thematic analysis was conducted followed by a coding-based “process of change analysis.” Participants were 50 young people in London schools who experienced moderate or severe emotional symptoms and had participated in up to 10 sessions of a school-based humanistic intervention. Participants were predominantly female and ethnically heterogeneous. Therapist qualities most often perceived as helpful were affiliative in nature. Unhelpful therapist activities were silences and a lack of input. Young people described feeling free to talk and open up. Helpful outcomes included feeling unburdened, gaining insight, and improving relationships. “Getting things off their chest,” “Advice and guidance,” “Modeling relationships,” and “insights to behavior change” were identified as specific processes of change in over 50% of young people. Approximately one-third felt hindered by a lack of therapist input, silences, or not feeling able to open up or trust. These findings indicate the potential value of an active, “process guiding” stance in humanistic therapy. Our process of change analysis has potential for identifying perceived change mechanisms in therapy. This work was supported by the Economic and Social Research Council [grant reference ES/M011933/1]. Anonymized qualitative interview transcripts are available on request to the First Author/Chief Investigator. Quantitative, participant-level data for the ETHOS study (with data dictionary), and related documents (e.g., parental consent form), are available via the ReShare UK Data Service (reshare.ukdataservice.ac.uk/853764/). Access requires ReShare registration.

## Highlights

- This study examined young people’s perceptions of what was helpful and unhelpful in humanistic therapy, and processes of change.
- Humanistic therapy may be helpful for young people by allowing them to get things off their chests, obtain advice, model positive relationships, and develop insights.
- Approximately one-third of young people wanted more input from their humanistic therapist, and a similar proportion found the silences awkward.
- Our findings indicate that an active, process guiding therapeutic stance can be perceived as particularly helpful by young people in humanistic therapy.
- A novel process of change analysis method can be used to reliably identify perceived cause-and-effect mechanisms in therapy.

Young people are particularly vulnerable to psychological difficulties (Blakemore, 2019). The World Health Organization (2021) reports that, globally, one in seven 10–19-year-olds meet criteria for a “mental disorder,” and that suicide is the fourth leading cause of death for 15–19-year-olds. Childhood disorders often continue into adulthood and can have longstanding social and economic consequences (Chen et al., 2006).

Educational settings may provide young people with unparalleled access to services; alleviating barriers such as time, location, and cost (Werner-Seidler et al., 2017). As a consequence, school-based services can increase young people’s use of mental health support (Kaplan et al., 1998) and reduce inequities in mental health care (Knopf et al., 2016). Worldwide, school-based therapy takes a variety of forms. In the United States, a cognitive behavioral therapy (CBT) approach is most prevalent, with a particular focus on educational attainment and career guidance (American School Counselor Association [ASCA], 2019). In the United Kingdom (UK)—as in several other regions of the world, such as Malta and Ghana (Harris, 2013)—school-based therapy primarily takes a *humanistic* form (Cooper et al., 2021; Copeland et al., 2024).

The term “humanistic” refers to a family of therapeutic approaches that share a core set of values and practices: a belief in the growth potential of the client; an emphasis on emotions; and a relational, phenomenological stance (Cain et al., 2016). Humanistic therapy draws extensively from the work of Carl Rogers (1959), who argued that relational factors were necessary and sufficient for positive change. This assertion formed the basis for Rogers’s *person-centered* approach which, today, is itself a family of practices (see Cooper, 2024). These range from a strictly “non-directive” approach (referred to as “classical” person-centered therapy) to more “process-directive” approaches, such as “emotion-focused therapy” (Elliott & Greenberg, 2021). In this respect, while humanistic therapy tends to be less directive than approaches such as CBT, it can include more process-directing elements: with the potential, for instance, for the therapist to introduce creative practices or relaxation methods. While humanistic therapy may be practiced alone, humanistic principles and methods can be used in combination with other approaches, such as CBT (e.g., Josefowitz & Myran, 2005). Indeed, humanistic practices such as empathy and unconditional positive regard are amongst the most widely used techniques across different orientations (Thoma & Cecero, 2009).

Humanistic therapy has been considered particularly appropriate for use with children and young people (Kelchner et al., 2019). Young people may value the choice and control that a client-led approach allows (Churchman et al., 2019), as well as the opportunity to express emotions in a supportive and trusting relationship (McArthur et al., 2016). In addition, as the humanistic approach is not diagnosis-centered, it may be particularly suitable for the wide range of psychological concerns presenting in an educational setting (e.g., bullying, bereavement, anxiety) (Cooper et al., 2021). Randomized controlled research indicates that school-based humanistic therapy can bring about large improvements in personal goal attainment for young people, and small to medium reductions in symptoms of psychological distress (Cooper et al., 2021; McArthur et al., 2013; Pearce et al., 2017). These effects are of a similar magnitude to other forms of school-based counseling and psychotherapy interventions (Baskin et al., 2010).

Despite such evidence of positive benefit, as with other therapeutic approaches, little is known about how change can come about for young people in humanistic therapy (Kazdin, 2009). Understanding the aspects of an intervention that bring about (or hinder) positive change—and the processes by which they do so—is important as it can be used to enhance intervention effectiveness (Cooper and McLeod, 2015; Fuertes & Nutt Williams, 2017). It allows researchers, trainers, and clinicians to build on those aspects found to be most helpful while minimizing those aspects found to be hindering. Such evidence can also help to develop an understanding, more broadly, of what is useful, and what is not useful, for particular client groups.

Determinants of change in psychotherapy can be studied through a variety of quantitative and qualitative methods (Krause, 2024), and from a range of perspectives (e.g., therapists, clients, and observers). In the field of adolescent therapy, much of the research has adopted a qualitative, self-report design: asking young people themselves, through interviews or questionnaires, what they perceived as the most (and least) helpful aspects of therapy, and how they perceived change as coming about. Such designs allow for in-depth insight into young people’s phenomenological experiences, and privileges those young people’s own perceptions. A qualitative meta-synthesis of such data from nine studies of predominantly humanistic approaches with young people found that, most commonly, young people said that it was helpful to talk and be listened to (Griffiths, 2013). The next most frequently identified helpful aspects were the therapist’s advice, getting things off one’s chest, and the therapist’s personal qualities (such as being “friendly”). These factors have also been identified as helpful by young people in other forms of psychotherapy, including psychodynamic (Bondi et al., 2006), cognitive-behavioral (Bru et al., 2013; Garmy et al., 2015; Herring et al., 2022; Lewis-Smith, Pass, Jones, et al., 2021; Wilmots et al., 2020), and integrative counseling approaches (Crocket et al., 2015; Gibson and Cartwright, 2014). Other frequently identified helpful aspects of therapy for young people, across both humanistic and non-humanistic approaches, are developing insight, problem-solving, acquiring new perspectives, and building self-esteem (Churchman et al., 2019; Goo et al., 2019; Housby et al., 2021). In short-term psychoanalytic psychotherapy, young people identified collaboration with their therapists as the most important factor in achieving good therapeutic outcomes (Housby et al., 2021). For young people in trauma-focused CBT, therapist authenticity and maintaining autonomy during sessions were identified as most important (Eastwood et al., 2021).

In terms of hindering aspects, young people in humanistic therapies most frequently said that they found it difficult to talk (for instance due to shyness, Griffiths, 2013). Other hindering aspects were a lack of therapist input, the demographic characteristics of the therapist (e.g., gender), and a perceived lack of confidentiality. Such difficulties in engaging with therapy have also been identified in the wider youth psychotherapy field (Herring et al., 2022). In CBT, some young people have found the emphasis on structure, exercises, and negative thoughts as hindering (Bru et al., 2013; Garmy et al., 2015; Herring et al., 2022). In addition, young people in brief behavioral activation found the brevity of therapy unhelpful (Lewis-Smith et al., 2021).

A study of psychotherapy outcomes for young people with depression accessing CBT, short-term psychoanalytic psychotherapy, or a brief psychosocial intervention found perceived helpful and hindering aspects differing across modalities (Krause et al., 2021). Qualitative interviews with 34 triads of young people, parents, and therapists found young people were more concerned with symptoms, self-management, and coping than their parents and therapists. Participants within the CBT group of the study were most concerned with symptoms and functioning. Within the psychoanalytic psychotherapy, personal growth was of highest concern.

Despite their value, studies of helpful and hindering aspects of therapy have several conceptual and empirical limitations. First the meaning of “aspects” or “factors” is often not well-specified, such that it may refer to in-session activities (by client, psychotherapist, and/or both); experiences of the intervention; and/or proximal in-session outcomes, intermediate post-session outcomes, or distal post-treatment outcomes (Hill et al., 2023). Second, some identified aspects (such as “talking and being listened to”) are vague and over-inclusive, making it difficult to apply to practice. Third, in several studies (e.g., Cooper, 2004), aspects of the intervention are coded as “helpful” or “hindering” simply by virtue of being perceived, by the researchers, as being of positive or negative valence respectively—but without the young person specifically indicating that these aspects have these particular effects (Cooper & McLeod, 2015). Related to this, helpful or hindering aspects are often identified as “stand alone” elements, without a clear indication of the specific mechanisms—if any—by which they might bring about change (Cooper and McLeod, 2015).

An alternative strategy for understanding what clients perceive as helpful and hindering in therapy is to focus on client-perceived *processes of change* (Cooper & McLeod, 2015; McArthur et al., 2016). This involves identifying the specific, perceived cause-and-effect pathways by which clients may attribute certain in-session activities as leading to certain outcomes. Such an analysis may provide a more robust indicator of client-perceived change because it requires specific in-session activities to be associated to specific outcomes, along with explicit assertion by the client that the former has led to something positive or negative.

In a first study aiming to identify processes of change in school-based humanistic counseling, McArthur et al. (2016) analyzed qualitative interview data from 14 young people. Using a grounded theory approach, they first categorized the data into (a) helpful factors and (b) positive changes (i.e., outcomes), and then identified instances in which the former were explicitly linked to the latter. “Relief” was found to be the most common self-perceived process of change: the release of a build-up of emotions (particularly anger or anxiety). Other identified processes of change were increases in self-worth through talking to a non-judgmental therapist, insight into self and other through being given an opportunity to reflect, the development of coping strategies through therapist guidance, and improved relationship skills through the modeling of healthy and open relating. Harrison (2020), directly coding change processes using thematic analysis, found similar pathways in a sample of 25 Chinese young people across various forms of therapy. Outside of humanistic therapy, thematic analysis indicated young people in brief behavioral activation felt more motivated to make decisions and develop self-awareness through understanding their own values (Lewis-Smith, Pass, Jones, et al., 2021; Lewis-Smith, Pass, & Reynolds, 2021).

The principal aims of the present study were to (a) establish the aspects of humanistic therapy that young people perceived as helpful and hindering, (b) test a novel means for identifying—in a robust, reliable, and quantifiable way—helpful and hindering processes of changes in therapy, and (c) establish what those helpful and hindering processes of change were for young people in humanistic therapy. To achieve these aims, we extended previous research in eight ways. First, we used a much larger sample than in previous studies (*N* = 50), so that we could detect lower frequency elements—particularly hindering ones, that are less commonly identified in the data (Griffiths, 2013). Second, we used a homogenous, clearly-defined sample, as compared with the heterogeneous samples that have characterized most previous research in this field. Third, all young people participated in the same manualized, adherence-checked form of humanistic therapy: school-based humanistic counseling. In only one of the previous studies had such adherence to the model been established (McArthur et al. 2016). Fourth, our interviews and analyses specifically focused on what young people described as “helpful” and “hindering,” rather than relying on post-hoc interpretations of the valences of experiences. Fifth, we made extensive efforts to follow up young people who had dropped out of therapy as well as completers, so that we were more likely to capture hindering aspects and processes. Sixth, in contrast to previous studies, we used multiple coders for our analyses; checking—and, where appropriate, working to enhance—inter-coder reliability. Seventh, so as to build on previous findings, our examination of helpful and hindering aspects included deductive design elements—establishing an a priori “logic model” and using this to inform parts of our interview schedule. Finally, as indicated above, we developed a novel procedure for attempting to identify, and quantify, processes of change, with the development of a coding manual and procedures to assess inter-rater reliability.

## Methods

### Design

This was a qualitative interview study with two phases of data analysis: (a) thematic analysis (TA, Braun & Clarke, 2006, 2022), and (b) novel *process of change analysis* (Cooper & McLeod, 2015; McArthur et al., 2016). TA is typically considered to consist of three main schools, namely “coding reliability,” “codebook,” and “reflexive,” which differ in their conceptual underpinnings (Braun and Clarke, 2022). Our approach to TA can be characterized as “medium q” (Clarke & Braun, 2018)—most closely aligned to the “codebook school” of TA (Braun & Clarke, 2022). Here, we aimed to combine the “Big Q TA” focus on rich and in-depth engagement with the data—diving “beneath the data surface” (p. 108)—with the structured coding procedures of “small q TA”, facilitating reliability and accuracy. Hence, our approach to TA combined inductive and deductive elements. Inductively, we aimed to provide the young people with an opportunity to express, freely, whatever was helpful and hindering to them, and to adopt a broadly “grounded” approach to data analysis (Glaser, 1995). In addition, reflexivity was undertaken to enable bracketing our a priori assumptions, allowing the data to drive the development of themes. Deductively, on the other hand, we also asked the participants to confirm or disconfirm whether specific aspects, previously identified in the literature as either helpful or hindering, were “true” for them, and divided the data into a priori domains.

A panel of young people (drawn from the Young Person’s Advisory Group at the National Children’s Bureau, NCB, a UK-wide children’s charity) and a panel of parents and carers (drawn from the Parent and Carers Advisory Group at NCB) advised on the development of methods. This included guidance on the choice of outcome measures, the development of participant-facing materials, and strategies for reducing the burden of the research on participants. Representatives from both panels joined the Trial Steering Committee, which met throughout the duration of the study, advising on all elements of study design, progress, and dissemination.

#### Study dataset

Data for this study were collected as part of a UK-based randomized controlled trial (RCT) of humanistic therapy for young people experiencing emotional symptoms (Cooper et al., 2021). A total of 329 young people (aged 13–16 years old) were randomized to either “school-based humanistic counseling” plus usual pastoral care (SBHC), or pastoral care alone (PCAU). The protocol for the trial is available at Stafford et al. (2018) and the statistical outcome findings are available at Cooper et al. (2021). The trial is registered with the ISRCTN Registry, number ISRCTN10460622. Ethical approval for the trial was obtained under procedures agreed by the University Ethics Committee of the University of Roehampton, Reference PSYC 16/227, 31^st^ August 2016.

### Participants

For the present qualitative study, we aimed for a total sample size of 50. Inclusion criteria for the trial were aged 13–16 years old and experiencing moderate to severe levels of emotional symptoms (as indicated by a score of 5 or more on the Emotional Symptoms subscale of the self-report Strengths and Difficulties Questionnaire, SDQ-ES, range = 0–10) (Goodman, 2001). Participants also needed an estimated English reading age of at least 13 years, a desire to participate in therapy, a school attendance record of 85% or greater (to increase likelihood of attending testing meetings), and not to be currently receiving another intervention. Exclusion criteria were: incapable of providing informed consent for therapy, planning to leave the school within the academic year, and deemed at risk of serious harm to self or others.

Participants for the full trial were recruited between September 29th, 2016, and February 8th, 2018, from 18 state-funded “secondary” schools in the Greater London area (typical age range 11–18 years old). We conducted 596 assessments for the trial and, in 330 (58.0%) cases, enrolled the young person (with one young person erroneously randomized twice, giving 329 participants). Qualitative interviews were conducted with a sample of young people from nine of the schools (*n* = 2–11 per school). Schools were selected to maximize representativeness across the full sample. In total, 53 young people assented to be interviewed (31.7% of all SBHC participants, 54.6% of young people in the nine schools). Of these, three interviews were unusable, primarily due to low sound quality, The final sample (*N* = 50) predominantly identified as female (88%) (which we address in our Discussion), with a mean age of 13.8 years old; 40% were of an Asian, African, or other minoritized ethnicity; and 56% had “very high” levels of psychological difficulties (Table 1). Compared with all SBHC participants, young people in the interview sample were significantly more likely to be female (χ^2^ = 9.7, *p* = .008), but were otherwise of a similar demographic profile.

**Table 1:** Baseline characteristics

	Interview Participants (*N* = 50)	All SBHC (*N* = 167)
Gender		
Female	44 (88%)	127 (76%)
Male	4 (8%)	37 (22%)
Other	2 (4%)	3 (2%)
Age (years)	13.8 (0.9)	13.7 (0.8)
Baseline Psychological Difficulties (SDQ-TD)		
Close to average	3 (6%)	20 (12%)
Slightly raised	11 (22%)	33 (20%)
High	8 (16%)	22 (13%)
Very high	28 (56%)	87 (52%)
School Year		
Year 8	8 (16%)	28 (17%)
Year 9	22 (44%)	79 (47%)
Year 10	18 (36%)	53 (32%)
Year 11	2 (4%)	7 (4%)
Ethnicity		
White	30 (60%)	90 (54%)
Asian/Asian British	7 (14%)	16 (10%)
African/Caribbean/Black British	4 (8%)	27 (16%)
Multiracial	9 (18%)	29 (17%)
Other	0 (0%)	4 (2%)
Missing	0 (0%)	1 (<1%)
Disability		
No disability	44 (88%)	142 (85%)
Has a disability	5 (10%)	23 (14%)
Missing	1 (2%)	2 (1%)

*SBHC* School-based humanistic counseling

#### Therapists

For the present qualitative study, the SBHC intervention was delivered by a pool of 10 therapists (one therapist per school, excepting one school that had two therapists). The therapists were recruited specifically for the purposes of the RCT and had not previously worked in the school to which they were assigned. Recruitment was undertaken by distributing a job advert and person specification to members of the largest professional body of therapists in the UK. Shortlisting and interviews were undertaken by two members of the research team (one academic and one trainer of therapy) and also included assessment by a panel of young people. Eight of the therapists were female, with a mean age of 44.8 years old (*SD* = 6.3, range = 25–63 years old). All of the ten therapists were of a white British ethnicity. All therapists were qualified to “diploma” level (at least a two-year, part time training in counseling or psychotherapy): seven on person-centered- or humanistic-identified training courses and three on integrative programs; all counselors had prior familiarity with the humanistic counselor competences framework (Roth et al., 2009). The therapists had been qualified for an average of 7.1 years (*SD* = 6.6, range = 1–25).

### Materials

The interviews conducted with the young people were semi-structured and based around a topic guide (Supplemental Material 1). The first, introduction section (~5 min), invited the young person to say something about themselves, why they thought they were offered therapy, and whether they had spoken to people in their lives about their problems. The second, open-ended section of the interview (~15 min), invited the young person to describe, in their own words, what they had found helpful or hindering in the therapy. To facilitate this, the young people were invited to fill out a blank “process map” (Supplemental Material 2). This consisted of rows of four empty ovals, linked together with arrows, in which the young people could write: “What the counselor did,” “How you responded to this,” “Any changes as a result,” and “What happened next” (43 young people completed at least one row of this map). The third, closed-ended section of the interview (~15 min), asked the young people to indicate if they had experienced helpful and hindering factors that had been previously identified in the literature, as reviewed by the trial team (Cooper et al., 2021; Logic Model, Supplemental Material 3).

### Procedures

#### Intervention

The young people were offered up to ten sessions of school-based humanistic counseling (SBHC) on an approximately weekly basis (Mean *N*_sessions_ = 8.0, *SD* = 2.4, range 1–10, *mdn* = 9). The therapists were instructed to practice according to the SBHC manual developed specifically for the trial (Kirkbride, 2016) and each therapist attended a minimum of five days’ training in this specific approach. The therapists were supervised on an approximately fortnightly basis by an experienced clinician (minimum of 6 years post-qualification).

SBHC is a discrete, manualized form of humanistic therapy. It is based on Rogers’s classical person-centered approach—prioritizing the development of a strong therapeutic relationship—but also allows for some degree of process direction, as considered appropriate to the individual client. The SBHC manual was based on evidence-based competences for humanistic therapy with young people aged 11–18 years (Hill et al., 2012). Therapist interventions prescribed in this manual centered on active listening; empathic reflections; and inviting young people to acknowledge, accept, and express underlying emotions and needs. Therapists were encouraged to form a positive therapeutic alliance with young people and to be active and alert: initiating, for instance, a collaborative assessment of the young person’s difficulties and therapeutic goals. Therapists were also instructed to consider the introduction of creative methods (such as drawing a picture) if it was felt that this could be helpful in the exploration and expression of the client’s emotions or situation.

All sessions were audio recorded, and adherence to SBHC—as set out in the manual—was assessed using the young person’s adapted version of the *Person Centred and Experiential Psychotherapy Rating Scale* (PCEPS-YP) (Ryan et al., 2023). The PCEPS-YP has nine items, with each item rated on a scale of 1 (showing none of this skill) to 6 (demonstrating this skill excellently). An example item, intended to measure empathic resonance, is “How well is the therapist able to resonate with, and communicate their understanding of, the young person’s spoken and unfeeling perceptions?” For each item, the PCEPS-YP includes supplementary guidance on behaviors that are good or poor demonstration of these competences. Psychometric analysis has demonstrated that PCEPS-YP shows a high level of internal consistency between scale items (α = 0.95). PCEP-YP total scores have also demonstrated moderate convergent validity (*r* = 0.37) with the Barrett-Lennard (2015) Relationship Inventory: a measure of therapist-provided interpersonal skills (Ryan et al., 2023).

There was a pool of eight auditors, all of whom were trained—or training—as humanistic therapists. All auditors attended one days’ training on the PCEPS-YP which included listening to and rating session recordings—previously calibrated by experts in the humanistic field. All eight auditors were assessed as showing high adherence to the calibrated ratings (<1.0 point divergence), and showed high correlations (*r* ≥ .9) between segment ratings (Cooper et al., 2021). Therapists’ adherence to SBHC was assessed independently by two of the eight auditors. Approximately 20-min audio segments (minimum segment length = 10 min) were randomly selected from an average of four (minimum of two) of the therapist’s randomly selected clients. The mean therapist adherence rating was 4.7 on the 6-point PCEPS-YP (*SD* = 0.3), with all therapists exceeding the pre-defined adherence cut-point, based on the PCEPS literature, of 3.5 (range: 4.2–5.1). This indicated that all therapists were assessed as practicing in line with the SBHC manual.

#### Interviews

The qualitative interviews were carried out on school premises, on average 5.5 weeks after the end of therapy (range: 1–16 weeks). There were four interviewers who carried out between two and 20 interviews each. The interviewers were researchers with extensive experience of qualitative research from the NCB. Transcription of the interviews was carried out by a professional transcription service, independent of the interviewers and data analysts.

### Qualitative Analysis

#### Thematic analysis

Thematic analysis for this study was conducted using NVivo v.11 and v.12 (detailed accounts of qualitative analyses, Supplemental Material 4), with coders blind to the young people’s demographic characteristics and outcomes. A preliminary organization of the data, drawing on Braun & Clarke’s (2006) six steps of TA, was conducted by Authors 2, 3, and 4. This organized the data into four higher-order themes: “expectations,” “experiences,” “responses,” and “outcomes.” An initial report of this analysis was reviewed by all authors. At this point, it was decided to re-analyze the data in a way that could answer, more directly, our TA research question (i.e., what was experienced as helpful and hindering). We therefore divided the data into eight a priori domains, each reflecting discrete elements of the therapeutic process identified in previous research and scholarship (e.g., Cooper & McLeod, 2011)—*therapist qualities*, *therapist activities*, *contextual qualities*, *client qualities*, *client responses*, *client activities*, *immediate outcomes*, *longer-term outcomes*—and established subdomains of *helpful* and *hindering* for each domain. Authors 1 and 5 then re-coded the interviews, inductively developing themes to populate, where relevant, each of the subdomains. This coding process progressed iteratively, with interweaving stages of independent coding, discussion, reorganization of the analytical frame, and reviewing of data coded into themes (example, Supplemental Material 5). Inter-coder agreement was established on 58 out of 74 coding units (78.4%). Authors 1 and 5 also undertook a self-reflexive exercise—writing down what they, themselves, had found helpful and hindering in their own therapy—as a means of recognizing, and facilitating the bracketing of, expectations and biases.

**Fig. 1:**
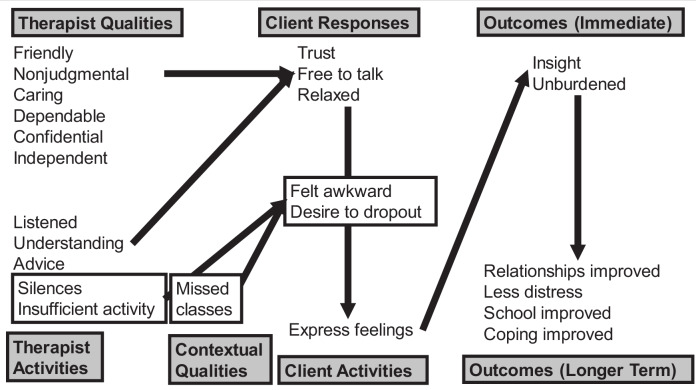
*Helpful and Hindering Aspects of School-Based Humanistic Counseling: Distillation of Most Prevalent Themes*. *Note*. Figure presents themes ≥50% participants for helpful, and ≥20% participants for hindering, across the whole interview. Boxed themes = hindering

The narrative and tabular presentation of our findings focuses on principal themes: defined as helpful aspects described by at least 25% of young people across both parts of the interview, and hindering aspects experienced by at least 10% of participants. Different cutpoints were used for helpful and hindering themes to compensate for potential deference effects and to ensure that the analysis paid sufficient attention to hindering aspects of the therapy as well as helpful ones. To distill this data into a more immediately readable format, the most prevalent principal themes are presented in Fig. 1 (helpful aspects ≥ 50%, hindering aspects ≥ 20%). A more extended table, with helpful aspects ≥10% of young people and illustrative extracts, is presented as Supplemental Material 6.

#### Process of change analysis

Alongside our TA, we wanted to see if it was possible to establish a robust and reliable new method for identifying, and quantifying, processes of change in therapy. This *process of change analysis* began with Author 1 creating *process of change narratives* for each of the 50 young people: summaries of what each young person concretely described as change processes in their therapy (example, Supplemental Material 7). To be coded as a helpful process of change, interview data needed to clearly link particular aspects of the intervention to particular positive in-session, post-session, or post-treatment outcomes, forming a coherent and intelligible chain of perceived cause-and-effect. For negative process of change, interview data needed to link particular aspects of the intervention to particular negative outcomes: again, in-session, post-session, or post-treatment. Commonly-identified helpful and hindering processes of change were written up, with descriptors, into a *Process analysis codebook* (Supplemental Material 8). A preliminary coding was then conducted of all 50 interviews by Author 1; with helpful and hindering processes rated for each young person. A second coder (Author 8) then independently coded ten of the interviews and worked with Author 1 to refine the *Process analysis codebook*. Two independent Master’s level students then carried out a full, independent coding of all cases (Authors 6 and 7), developing a modified coding scheme whereby processes were rated as being either “Present” or “Not present.”

## Results

### Thematic Analysis

In terms of principal themes, young people described as helpful their therapists’ friendliness, lack of judgment, care, dependability, confidentiality, and independence from the school; as well as their therapists’ activities of listening, understanding, and offering advice (Fig. 1). As a result of such factors, the young people felt trust towards their therapists, free to talk, and relaxed; and this could lead to expression of feelings. However, the young people could also feel hindered by their therapists’ silences and lack of activity, as well as by fears of missing classes. Consequently, young people could also feel awkward in the therapy and want to drop out. Immediate outcomes of the therapy were greater insight and a feeling of being unburdened; with longer term outcomes including improved relationships, less emotional distress, improvements at school, and better coping strategies.

#### Therapist qualities

##### Helpful

For the young people, the most helpful therapist characteristics were at the affiliative end of the interpersonal behavior spectrum (Table 2). Most commonly, this was therapists being *friendly and welcoming* (*n* = 47), with descriptions of the therapist’s warmth, niceness, kindness, and familiarity; and that they smiled a lot. The therapists were also described as being *non-judgmental and unconditionally accepting* (*n* = 44). For instance, one young person said, “I felt like I could say whatever I wanted without her having an opinion straight away.” Closely related to this, the young people described the therapist’s *caring* as helpful (*n* = 37): that the therapist had a genuine interest in them, and that their lives and problems seemed to matter to them. One young person, for instance, said, “She just made me feel like it wasn’t just like part of her job and she was waiting to get out really, she was happy to be there and talk with me.” Three other frequent responses were that it was helpful that the therapist was *dependable, reliable, and consistent* (*n* = 37); *confidential* (*n* = 36); and *independent from the school* (*n* = 27).

**Table 2:** Thematic Analysis: Domains, Subdomains, and Themes

DOMAIN *SUBDOMAIN* Theme	Total (open-ended + closed ended) *n* participants (%)	Open-ended only *n* participants (%)	Not experienced/not helpful *n* participants (%)
**THERAPIST QUALITIES**			
*HELPFUL*			
** Friendly and welcoming**	**46 (92%)**	**10 (20%)**	**3 (6%)**
** Non-judgmental and unconditionally accepting**	**44 (88%)**	**12 (24%)**	**3 (6%)**
** Caring**	**37 (74%)**	**10 (20%)**	**8 (16%)**
** Dependable, reliable, and consistent**	**37 (74%)**	**5 (10%)**	**24 (48%)**
** Confidential**	**36 (72%)**	**3 (6%)**	**10 (20%)**
** Independent from the school**	**27 (54%)**	**4 (8%)**	**13 (26%)**
*HINDERING*			
Over-friendly	5 (10%)	1 (2%)	
**THERAPIST ACTIVITIES**			
*HELPFUL*			
** Listened**	**48 (96%)**	**27 (54%)**	**4 (8%)**
** Understanding and empathic**	**43 (86%)**	**14 (28%)**	**8 (16%)**
** Advice and guidance**	**39 (78%)**	**24 (48%)**	**18 (36%)**
** Helping the client express feelings**	**24 (48%)**	**3 (6%)**	**12 (24%)**
Creative, artistic, and written media	19 (38%)	10 (20%)	
Questions	18 (36%)	10 (20%)	
Not forcing or rushing the young person to talk	16 (32%)	6 (12%)	
*HINDERING*			
Silences	13 (26%)	9 (18%)	
Insufficient activity: advice, guidance, strategies, activities, questions	12 (24%)	5 (10%)	
Guidance, activities	6 (12%)	4 (8%)	
**CONTEXTUAL QUALITIES**			
*HINDERING*			
Missed classes	11 (22%)	2 (4%)	
Not enough sessions	5 (10%)	0 (0%)	
**CLIENT QUALITIES**			
*HINDERING*			
Dislike talking, shyness	8 (16%)	6 (12%)	
**CLIENT RESPONSES**			
*HELPFUL*			
** Felt trust**	**40 (80%)**	**4 (8%)**	**8 (16%)**
Felt free to talk and open up	35 (70%)	18 (36%)	
Felt comfortable, relaxed, and not judged	28 (56%)	16 (32%)	
Felt happy, supported, and cared for	22 (44%)	17 (34%)	
Greater self-reflection, different perspectives	18 (36%)	11 (22%)	
*HINDERING*			
Felt awkward, uncomfortable, or weird	13 (26%)	9 (18%)	
Felt like they wanted to drop out or did drop out	10 (20%)	9 (18%)	
Felt they had not had enough to talk about	5 (10%)	3 (6%)	
**CLIENT ACTIVITIES**			
*HELPFUL*			
** Express feelings, get things off their chest**	**47 (94%)**	**26 (52%)**	**5 (10%)**
Took advice	14 (28%)	10 (20%)	
*HINDERING*			
Didn’t open up	7 (14%)	4 (8%)	
**IMMEDIATE OUTCOMES**			
*HELPFUL*			
** Greater insight and self-understanding**	**34 (68%)**	**8 (16%)**	**6 (12%)**
** Unburdened, relieved, a “weight” lifted**	**32 (64%)**	**14 (28%)**	**5 (10%)**
Calmer, less anxious, and more positive	23 (46%)	18 (36%)	
Feeling there was someone to talk to	20 (40%)	9 (18%)	
*HINDERING*			
More negative feelings and behaviors	6 (12%)	5 (10%)	
**LONGER-TERM OUTCOMES**			
*HELPFUL*			
** Improvement in relationships**	**42 (84%)**	**24 (48%)**	**24 (48%)**
** Reductions in emotional distress**	**35 (70%)**	**23 (46%)**	**7 (14%)**
** Improvements at school**	**34 (68%)**	**16 (32%)**	**16 (32%)**
** Improved coping strategies, resilience, and self-control**	**29 (58%)**	**11 (22%)**	**5 (10%)**
** Increased self-acceptance**	**27 (54%)**	**3 (6%)**	**10 (20%)**
Improved confidence, and self-esteem	19 (38%)	5 (10%)	

**Embolden themes** were specifically enquired into in the closed-ended part of the interview. Cutpoint for inclusion: Helpful aspects ≥ 25% of young people, hindering aspects ≥ 10% of young people, across both parts of the interview

##### Hindering

Five of the young people described it as unhelpful that the therapist was *over-friendly* or “overly-nice.” This was for a range of reasons. For two young people, it was because the therapist seemed, at times, “weird”; or as if they were putting on “an act.” One of these young people said: “I was like, ‘What’s up with her?’ Like, ‘Hmm, she doesn’t come from [local area], that’s for sure’.” For one young person, the therapist’s friendliness meant that they “strayed off from what we were supposed to be doing.” One young person described the therapist’s over-friendliness as unhelpful because they, then, felt that they also needed to put up a “mask” of friendliness.

#### Therapist activities

##### Helpful

In terms of what the therapist did that was helpful, the most commonly-endorsed response, given by nearly all the young people (*n* = 48), was that the therapist *listened*. One young person, for instance, said, “She wasn’t, like, doing something else. She was sat there all the time just like listening to me.” A number of the young people contrasted this with other adults, professionals, or friends in their lives who, they felt, did not (or had not) really attended to what they were trying to say.

Closely related to feeling listened to, a large majority of the young people described it as helpful that their therapist responded to them in *understanding and empathic* ways (*n* = 43). One young person, for instance, said, “They just talked to me like I was a human being not like, ‘Oh, you know, what teenagers are like. Boys! Dramatizing everything. 13 years as well, am I right?’ It wasn’t anything like that, it was very understanding which was cool.”

Third (and despite the relatively non-directive nature of the intervention, see Discussion) the young people described the *advice and guidance* they experienced from the therapist as helpful (*n* = 39). For 22 of the young people, this was in relation to relationship issues, such as encouragement to talk more to parents or friends, or to ignore bullies. For 14 of these young people, the advice involved ways of coping with stress and anxiety, such as “coping methods with panic attacks.”

A fourth helpful therapist activity was the therapist *helping the client express feelings* (*n* = 24). For instance, one young person said, “I’d speak a bit [about] something and they’d ask, ‘How do you feel about this? Do you feel distressed? Do you feel upset?’ I did express how I felt with these problems and that helped because then they’d understand how I felt about these problems.”

The use of *creative, artistic, and written media*—including drawing, playing games, role-playing, and doing worksheets—was described as helpful by 19 of the young people. In some instances, this was due to its immediate effects in the therapy: helping the young person calm down, or breaking uncomfortable silences. In other instances, it was seen as leading to longer term effects, in particular insights about emotions: for instance, through using a “color wheel” to identify and describe feelings.

For 18 of the young people, the therapist’s *questions* were experienced as helpful, particularly regarding how the young person felt about things. This was often in the context of other helpful relational therapist activities, such as listening, understanding, reflecting, and remembering. For instance, one young person said, “It was like they were taking in what I was actually saying and then the questions afterwards were based upon that.”

*Not forcing or rushing the young person to talk*, but allowing them to open up in a relaxed, calm, and non-pressuring atmosphere was described as helpful by 16 of the young people. One young person said, “He wasn’t trying to drag stuff out of me, so I wasn’t so guarded, and I was just like handing it to him on a plate.”

##### Hindering

Thirteen of the young people described the therapist’s *silences* in the therapy as unhelpful, expressing a wish that the therapist had spoken more. One young person said:

She didn’t have a lot to say. She would just sit there and stare at me for sometimes three minutes at a time. Literally it was three minutes. She’d just sit there, and I’d be looking around the room, and every time I looked back at her, she’s just looking at me. She wouldn’t say anything.

Closely related to this perceived lack of verbal therapist utterances, 12 of the young people described as unhelpful the therapist’s *lack of active input*. This included limited levels of “advice,” “guidance,” “strategies,” “feedback,” “questions,” and “opinions.” One young person said, “I just felt like, ‘Well, we’re talking—which is good, because I’m getting things off my chest. But if I’m not getting anything back from it such as advice or strategies or another way to go, then I found that bit useless’.”

Conversely, for a small proportion of young people (*n* = 6), it was the therapist’s active *guidance and activities* that were unhelpful. One young person said:

[The therapist] would do breathing exercises and imagine things, and I didn’t like that. I said, “I didn’t like that,” and [the therapist] was like, “Well, let’s just try.” “I don’t want to do it.” “Well, let’s just try.” [The therapist] was kind of persistent on something that I didn’t want to do.

#### Contextual qualities

##### Hindering

Eleven of the young people said it was unhelpful that, as a consequence of the therapy, they *missed classes*. This was particularly the case if it was classes that the young people liked; were struggling with; or were “core” subjects, like English and mathematics. For five of the young people, it was unhelpful that they had *not had enough sessions*: they wished that they had had more.

#### Client qualities

##### Hindering

For eight of the young people, a factor that was unhelpful to the therapeutic process was that they were *shy and disliked talking*. One young person, for instance, said, “I don’t like talking to a stranger”; while another young person described themselves as “not a very open person”; and a third young person said that they were “just naturally” someone who “keeps it in.”

#### Client responses

##### Helpful

As a consequence of the helpful therapist and contextual factors, the young people experienced a range of positive responses. Most frequently, the young people said that they *felt trust* (*n* = 40). This was spontaneously mentioned by only a small number of young people, but strongly endorsed in the closed-ended part of the interview. For the young people, experiencing trust was closely linked to feeling that they could open up. One young person said, “if you’re having counseling and you don’t trust the person, you feel like you can’t tell them stuff. But if you trust the person, you feel like you can tell them anything.” As with feeling comfortable, a proportion of these young people said that a sense of trust developed over the sessions (*n* = 13).

Many of the young people also said that they *felt free to talk and open up* (*n* = 35): particularly about feelings, as well as things that were upsetting them and problematic situations. Here, several participants said that they felt they could express whatever they wanted to the therapist: “things that I haven’t really spoken about to anyone before.” Another young person said, “the stuff that I didn’t feel comfortable saying to my parents I felt comfortable saying in the room.”

In total, 28 of the young people said that the therapist activities meant that they *felt comfortable, relaxed, and not judged*. One young person contrasted this with the normal school environment, in which “everyone is judging everyone, really. So it doesn’t matter if you’re tall, skinny, they’ll always just judge. Then for someone [the therapist] not judging, it made me feel more comfortable and more welcome.”

The young people also described experiencing a positive affective state as a result of the therapists’ activities: *felt happy, supported, and cared for* (*n* = 22). Young people said, for instance, that being listened to and understood put them in a “better mood” or that the therapist’s guidance made them feel like they had “someone behind me.”

A final helpful response that the young people described was *greater self-reflection, different perspectives* (*n* = 18). Here, the young people described feeling that, through the therapists’ activities and qualities, they had time to think and reflect on things: to ask, “‘Oh, how am I feeling right now?’” It also referred to coming to see their issues from different viewpoints. One young person said, “They’ve made me think about things that I was going through from a different perspective which was really cool.”

##### Hindering

In terms of negative responses to the therapy, 13 of the participants said that they *felt awkward, uncomfortable, or weird*. This was primarily in response to the therapist’s silences. One young person said:

sometimes there’d be points where– after I’d said something, he would just stare at me, and there would be an awkward silence for ten seconds and he’d be like, “Oh…” Then, I’d be like, “I don’t really know what else to say.” He would be like, “Do you just want to finish now?” or something, so it was a bit awkward at times.

However, some participants also felt awkward in response to a lack of input, from opening up sensitive areas, or from feeling that the therapy was “cheesy.” With respect to this last issue, one young person said:

[The therapist] did do this really awkward thing that made me feel very uncomfortable and not want to come anymore. She’d like– I would speak and I would like, “Yeah.” She goes, “Hmm, yeah. Yeah, hmm. Yeah.” She just kept on saying, “Hmm, yeah. Hmm, yeah.” I was like, “Hmm.” So it was really awkward, and like the room was silent, and everybody’s sitting there going, “Hmm.”

As with this young person, 10 of the young people said that, at some point, they had *felt like they wanted to drop out or did drop out* of therapy. For five of the young people, this was because of the awkwardness they experienced. One young person said, “I was like, ‘Och, I can’t deal with more awkward stuff. I’m too awkward myself.” However, in the two cases where the young people did, actually, stop going to therapy, it was because they were concerned about missing key lessons. Two of the young people said that, although they wanted to drop out, they did not do so because they felt bad for the therapist.

As a third unhelpful response, five young people said that they *felt they had not had enough to talk about*: running out of things to say during the sessions. One young person said:

I didn’t really have that big problems, it would all be about the same thing every week. I think he got a bit tired about talking about it. It was always about school, or my family. I don’t know. It’s just a bit boring I think.

#### Client activities

##### Helpful

Nearly all the young people said that, as a result of the above factors, they could then go on to *express feelings, get things off their chest* (*n* = 47). This was a process of “opening up,” of “letting things out,” particularly emotions, stressors, and previously unexpressed thoughts and feelings. One young person said, “you’ve got all the negativity inside of you and you’ve got the positive inside of you but you’re drawing all the negativity out because you’re being able to express the negativity to someone else.”

Second, the young people said that they *took advice* from the therapist, implementing the guidance and suggestions that had been offered to them (*n* = 14). One young person gave the following example:

I used to have an argument with this girl…and the therapist said to just leave it because she’s just going to– she wants attention and she wants a reaction out of me. She [the therapist] was just like, “Leave it. She can say whatever she wants and then she’ll stop it herself,” and now that girl doesn’t even say anything to me because I don’t even say anything to her.

##### Hindering

As the opposite of express feelings, seven of the young people said that, at least to some extent, they *didn’t open up* in the therapy. They described, for instance, “keeping things in,” becoming more “guarded,” or telling the therapist that they did not want to talk about certain things.

#### Immediate outcomes

##### Helpful

The most frequent outcome, both proximal and extending into the longer-term, was *greater insight and self-understanding*, including the development of new perspectives on self and other (*n* = 34). One young person, for instance, described learning the “triggers” for their anger and what they could do to prevent them. Another young person said that, by telling the therapist about their friendship group, they realized more who they should be spending time with.

As a consequence of expressing feelings and opening up, the young people also described feeling *unburdened, relieved, a “weight” lifted* (*n* = 32). One young person, for instance, said, “little things kind of build-up, and I could just say [in the therapy] if someone annoyed me, I could be like, ‘This person just annoyed me today,’ and that’s it, it’s out of my mind, it’s not like nagging.”

The young people also described the therapy as leading to feeling *calmer, less anxious, and more positive* (*n* = 23). Here, the young people described coming out of the therapy “in a nicer mood,” “happier,” more “peaceful,” less “stressed,” and less “angry.”

As part of these immediate, proximal impacts of the therapy, 20 of the young people described the prospective benefits of *feeling there was someone to talk to*. This referred to the young people’s knowledge that they would be talking to the therapist during the week, and the sense of relief and comfort that came from that: “she was there for me if I needed her”.

##### Hindering

The one hindering in-session and post-session outcome, described by six of the participants, was *more negative feelings and behaviors*. One young person, for instance, said that the one thing they did not like about the therapy is that they would come out of the sessions feeling more “stressed,” “angry,” and “snappy”; and that there would be more arguments at home on those days. For another young person, “It’s just when you talk about all the bad stuff, it just makes you feel a bit bad, as well.”

#### Longer-term outcomes

##### Helpful

The most commonly-reported distal outcome was *improvement in relationships* (*n* = 42). Improvements were particularly with parents and carers, other family members, and friends. The young people described getting on better with others and being closer; having less arguments and fights; being kinder, nicer, and more empathic; and opening up, and trusting, more. The majority of these improvements fitted into a sub-theme of better communication (*n* = 36); and a principal sub-sub-theme here was that opening up to the therapist led to opening up to other significant people (*n* = 26). For instance, one young person said:

I think expressing my feelings has always been difficult for me, but I felt comfortable enough to just say as it was with him [the therapist], and I thought, “Well, if I can say it to a complete and utter stranger why can’t I not say to a family member,” because a stranger is more likely to turn around and run in the opposite direction than a family member?

*Reductions in emotional distress* was a second, longer-term outcome (*n* = 35). Most commonly this involved feeling less “sad,” “moody,” and “happier” (*n* = 24). Other reductions in distress were less “anxious,” “stressed,” “worried,” and “calmer” (*n* = 17).

*Improvements at school*, as a consequence of therapy, were described by 34 young people. The most frequent subtheme here was a greater ability to concentrate, or focus, in class (*n* = 18). This was often related to getting things off their chest. One young person said:

Once I’d get something off my chest, I’d go back to lessons and I wouldn’t feel so heavy weighted on subjects that didn’t really apply to school. My school work would be so much easier for me to look at and go, “Yes, I can do that,” rather than thinking about things that I didn’t need to think about.

A fourth set of longer-term positive outcomes involved *improved coping strategies*, *resilience, and self-control* (*n* = 29). This included learning methods to deal with stress, anxiety, and panic (such as sport); and learning to think situations through—or walk away from them—rather than responding reactively.

*Increased self-acceptance* was described by 27 young people: less judgmental and critical about their own selves, feelings, and behaviors. One young person said, “before I started counseling I didn’t like anything about myself, I thought I was just useless; and now I would say, ‘Yes, I’m not perfect but I can improve’.” Closely related, 19 of the young people said the therapy led to *improved confidence and self-esteem*. One young immigrant, for instance, said that therapy helped them feel more confident to be themselves in the UK, and not worry so much about making language mistakes. Increases in confidence were often attributed to being listened to, and accepted, by the therapist, such that the young people came to value their own voices.

### Process of Change Analysis

#### Inter-rater reliability

Averaging across all phases, inter-rater reliability for coding of the processes of change (Cohen’s Kappa) was at acceptable levels: ranging from 0.69 for “Getting things off their chest” to 0.95 for “Silence awkward” (see Supplemental Material 9). The median inter-rater reliability was 0.84.

#### Helpful processes of change

We identified seven helpful processes of change. The most commonly identified of these was *Getting things off their chest* (*n* = 37, Table 3). This was followed by *Advice and guidance* (*n* = 33), *Modeling relationships* (*n* = 28), *Insight to behavior change* (*n* = 26), and *Developing self-worth* (*n* = 24). Less commonly, young people also described *Awareness of support* (*n* = 11) and *Learning creative methods* (*n* = 5).

**Table 3:** Helpful Processes of Change

*Change process* Summary	Example	*n* (%)
*Getting things off their chest* Unloading problems during the sessions made YP feel a sense of relief, reduced stress, and concentrated more	“I had a lot of stress. I carry a lot of stuff with me, and it helped get a lot of the stress off.”	37 (74%)
*Advice and guidance* Coping strategies and guidance from T helped YP form good interpersonal relationships	“He said to always tell someone if I’m having a bad day or be open with my feelings and support each other…Yes, because, as I said, my mum and my dad are there for me now.”	33 (66%)
*Modeling relationships* Experiencing an open and positive relationship with T enabled YP to open up to other people in life	“At the start, I didn’t really trust a lot of people outside of counselling and then I started to have a different perspective on other people, so obviously I had a lot more trust built up.”	28 (56%)
*Insight to behavior change* YP understood and learnt better about themselves, leading to a behavior change	“I realized that if I have a problem, I should state it….Yes, because if I had an argument– well not an argument, but a misunderstanding with my friends, then I would talk to them and be like, ‘Sorry, I’m just having a bad day,’ and they would understand more.”	26 (52%)
*Developing self-worth* Being accepted or listened to or positive feedback from T helped YP feel better about themselves and express themselves positively	“I feel more confident in myself and I keep telling myself, ‘It’s okay to feel that way, you’re fine, you’re good,’ because I get very anxious around people. It’s like they’re judging you, they’re looking the way– it was like, ‘Oh, my hair’s looking this way, oh no, my makeup is looking that way,’ or ‘I’m not dressed right,’ I always have this thing and she [the therapist] is like, ‘It’s fine, it’s normal, it’s okay,’ so, yes.”	24 (48%)
*Awareness of support* An awareness that seeing T later during the week led the YP to feel less anxious	“Well, I guess, when I had something on my mind, I’d be like, ‘Oh, I have counselling this Thursday. I can go talk about it there.’”	11 (22%)
*Learning creative methods* Using creative methods helped YP to take these strategies to manage their feelings outside the sessions	“So now I’ve got the ‘fidget spinner’ that I use….So I always have something to do when I’m writing as well which is even better….what I have now is really helping me to concentrate more because once I’m doing the spinning thing, I can listen to what the teacher is saying as well.”	5 (10%)

#### Hindering processes of change

We identified five hindering processes of change. The two most frequent were *More input wanted* (*n* = 15, 30%) and *Silences awkward* (*n* = 14, 28%) (Table 4). Clients also identified *Can’t open up/trust* (*n* = 13, 26%), *Unnatural/clichéd* (*n* = 8, 16%), *Feel worse* (*n* = 7, 14%), and *Miss lessons* (*n* = 7, 14%).

**Table 4:** Hindering Processes of Change

Change process Summary	Example	*n* (%)
*More input wanted* YP needed more advice, feedback, and strategies from T	“I feel like I wasn’t able to, like to get feedback.”	15 (30%)
*Silences awkward* Awkward silence during the sessions made the YP uncomfortable or want to discontinue the sessions	“She didn’t have a lot to say. She would just sit there and stare at me for sometimes three minutes at a time.”	14 (28%)
*Can’t open up/trust* YP reported that it is challenging to trust or open up to T	“I feel like some things I could but not for everything. Like, not because of her, but because of me, and that I don’t really trust many people.	13 (26%)
*Un-natural/clichéd* YP described T or sessions as unnatural, weird, or clichéd	“Sometimes she’d say things like, ‘Oh, I was thinking about you this weekend, blah, blah, blah.’ I was like, ‘Why were you thinking about me over your weekend?’ It was a little bit weird.”	8 (16%)
*Feel worse* YP reported feeling upset or worse after the sessions	“I became a bit more guarded, just like he didn’t listen… It put me in a bad mood… About the rest of the week.”	7 (14%)
*Miss lessons* YP reported missing lessons because of therapy sessions or felt anxious, or had other adverse effects due to not attending lessons	“I stopped going because I was missing out on the key lessons that I had to go to, like the ones that I was struggling in, and then I would just go back into the lesson and I wouldn’t understand what they were doing.”	7 (14%)

*T* therapist, *YP* young person

## Discussion

Our TA of helpful and hindering aspects of humanistic therapy provided a rich, multifaceted, and comprehensive description of the different elements that appeared facilitative and obstructive in this work. Our findings provide strong support to previous analyses (Griffiths, 2013), indicating that young people in humanistic therapy value a friendly, non-judgmental, caring, and empathic listening relationship in which they can express their feelings, and develop insights into self and others. Although the particular therapy used in our study was humanistic and with a predominantly female sample, previous research on helpful and hindering aspects of therapy with young people (e.g., Eastwood et al., 2021; Krause et al., 2021; Lewis-Smith et al., 2021) suggests that these processes may be relatively ubiquitous across therapeutic orientations.

The procedures we developed, and tested, for our process of change analysis appeared relatively successful. Not only were we able to identify specific, perceived cause-and-effect pathways from the young people’s narratives, but the results of this analysis corresponded to the themes identified in a more established TA. Most importantly, we achieved adequate to high levels of inter-rater reliability across processes of change. Working independently, coders could identify similar processes of change in young people’s narratives. While the results of this process of change analysis, to a considerable extent, overlapped with the results of our TA, the former procedure has several advantages: establishing, not just the aspects of therapy that are perceived as helpful and hindering; but the specific, self-perceived processes through which these aspects may lead to specific proximal, intermediary, and distal outcomes. This may help to overcome some of the vagaries of helpful/hindering aspects research. In addition, our coding procedure allowed for clear and meaningful tests of inter-rater reliability, with quantified outcomes that could be used for a wide range of subsequent process–outcome analyses (see, for instance, Supplemental Material 10). We discuss these possibilities under further research, below.

In terms of the processes of change identified in our predominantly female sample, our findings support previous research (e.g., McArthur et al., 2016) that “Getting things off their chest,” or “Relief,” is one of the most commonly experienced processes of change by young people in humanistic therapy. Qualitative meta-analysis indicates that such a process of “Experiencing relief” (Ladmanová et al., 2022) is also commonly reported by adults as an impact of helpful events in psychotherapy. In addition, our study supported McArthur et al. (2016) and Harrison’s (2020) findings that “Insight to behavior change,” and “Developing self-worth,” were commonly-reported processes of change in humanistic therapy for young people. The first of these has been identified in other therapeutic approaches with young people (Housby et al. 2021; Lewis-Smith, Pass, & Reynolds, 2021), and is the most commonly identified change process in adult psychotherapy: “Gaining a new perspective on the self” (Krause, 2024; Ladmanová et al. 2022). As with McArthur et al. (2016) and Harrison (2020), we also identified improved relational skills as an outcome of humanistic therapy with young people. Additionally, the development of improved relational skills through modeling in the therapeutic relationship could be distinguished from their development through direct guidance and psychoeducation.

Interestingly, none of the self-identified processes of change identified in our predominantly female sample mapped specifically onto the Rogerian model of development at the heart of humanistic theory. According to Rogers (Cooper, 2013; Rogers, 1951, 1959), an unconditionally accepting, empathic, and genuine therapeutic relationship allows the client to embrace the totality of their “organismic” experiencing. As a consequence, they become more connected with their actualizing tendency (the “organismic valuing potential”) and thereby more likely to act in ways that are genuinely self-maintaining and/or self-enhancing. Although several of our processes of change had elements of this mechanism, few of our young people directly referred to feeling more “themselves” or more attuned to their genuine feelings or needs. However, absence of this mechanism should not be taken as evidence that it did not occur, only that it was not reported within the study—perhaps because young people find it difficult to articulate their emotions to others (Wylie et al., 2023). Literature suggests that adolescents are highly motivated towards developing authenticity and understanding their genuine self (Thomaes et al., 2017) and that this process can be supported within a therapeutic setting that offers satisfaction of autonomy (Alchin et al., 2024).

Our second most frequently self-identified helpful process of change, “Advice and guidance,” would seem to directly contradict Rogers’s (1961) classical assumptions about change in therapy: “It is the client who knows what hurts, what directions to go, what problems are crucial, what experiences have been deeply buried” (pp. 11-12). However, this valuing of advice and guidance is ubiquitous across the research on school-based humanistic and person-centered therapy with young people (e.g., Cooper, 2004; Griffiths, 2013; McArthur et al., 2016); and, indeed, in the wider literature on therapeutic interventions for young people and adults (e.g., Bjornestad et al., 2018). One possible explanation here is that what our predominantly female sample perceived as “advice” was, in fact, empathic reflections of their own experiences and perceptions. However, in many cases, the advice that the young people described receiving—such as breathing methods for reducing stress or strategies for being more assertive—seemed too technical and specific to have been self-generated. Furthermore, closely connected to this, around one-third of the young people said that they found a lack of input, feedback, and strategies unhelpful; along with awkward and uncomfortable silences—a finding replicated within other therapies for youth (Housby et al., 2021). For our humanistic intervention, metacompetences included, “an ability to maintain a balance between directive and non-directive dimensions of the therapeutic process, as appropriate to the individual client” (British Association for Counseling and Psychotherapy, 2022, p. 110). Our findings emphasize the importance, at least to a significant minority of young people, of these more directive elements. Clinical implications will be discussed below.

We found that approximately one-quarter of the predominantly female sample identified their own unwillingness to open up and trust the therapist as an obstacle to positive change. This is consistent with the well-established psychotherapy research finding that client factors (such as levels of motivation) are the principal determinants of therapeutic growth (e.g., Bohart & Tallman, 1999; Bohart & Wade, 2013). Such wariness towards therapists is also consistent with theories of adolescence, where a hypersensitivity to judgments from others has been found to exist (Bluth & Blanton, 2014), and a bias towards judging the intentions of others as negative (Bird et al., 2018). Difficulties in talking and disclosing have been identified previously in the adolescent therapy literature (Griffiths, 2013)—though also with adults (Ladmanová et al., 2022). Trust is also becoming a more central focus of psychotherapy theory and research (e.g., Allen, 2022), with Wilmots et al. (2020) discussing its centrality to the development of the therapeutic relationship in CBT with depressed adolescents. Although our research method did not allow for a cross-case comparison, young people who did not tend to find the intervention helpful were often the ones who, at endpoint interview, described themselves as generally distrustful of others, and that this relational stance had been extended to their therapist. In a few instances, this distrust was described as attenuating as the therapy progressed; but, in most cases, it was not, and led to poorer outcomes or a desire to dropout. These findings point to the role that young people’s trait-like relational and attachment styles may have in moderating therapeutic outcomes (e.g., Saatsi et al., 2007). However, therapist qualities and activities—such as expressing warmth, care, and professionalism (Wilmots et al., 2020)—may also have an important role in establishing the conditions in which young people’s trust can emerge.

Overall, our findings are consistent with a “pluralistic” understanding of change processes in therapy, as multiple, heterogeneous, and individualized: that both relational and technique-based factors can contribute to positive change, with considerable variation at the individual level (Cooper & McLeod, 2011). Consistent with a common factors approach (e.g., Wampold & Imel, 2015), our findings would also support the claim that many change processes may be transtheoretical, with no clear one-to-one relationship between what therapists are primarily trying to do and what clients may actually perceive as helpful or hindering.

In terms of limitations, our study relied on self-perceived and self-reported processes of change. Our young people may therefore not have been aware of, or may have misreported, the actual mechanisms of change in their therapy. The self-report nature of our study also means that it was vulnerable to a range of demand characteristics, such as young people wanting to present themselves as “good clients” who have shown positive change. Our results, therefore, provide only a partial picture of what may be driving change in SBHC—and therapy more generally—and one that needs triangulation from more external, objective sources. Nevertheless, qualitative data may have a particularly valuable role in establishing causality and understanding each element in a process of change: providing a medium through which participants can describe the specific, generative effects of in-session incidents (Higginson & Mansell, 2008; Maxwell, 2012).

A major limitation of our study was the disproportionate numbers of female participants. Although, in the UK, females are more likely to attend secondary school counseling than males at a ratio of about 60/40 (Cooper, 2009), our participants were 88% female. This seems, in part, due to more females coming into the main trial but, above this, significantly more females then agreed to be interviewed. Gendered role expectations may, in part, account for these differences, with males more reluctant to express their feelings and vulnerabilities (Bem, 1981). We did consider restricting our analysis to females alone. However, we felt that such an approach would have altered, post hoc, our original sampling frame; and removed, from our analysis, the perceptions of those who did not identify as female. In addition, our findings are generally consistent with those where more gender-balanced samples have been used (see, Griffiths, 2013); and in just one of 13 post hoc point biserial correlations did we find significant differences between self-identified processes of change in females versus non-females (Supplemental Material 10). Nevertheless, we must be cautious in generalizing from our results to young people of all genders: for instance, young females may be more likely to value relational processes than males (Bem, 1981).

Other limitations of our study were that we looked at just one form of therapy; and that the sample was based in just one region of the UK, where levels of mental disorder are particularly high (Knowles et al., 2021). Our findings, therefore, may not be generalizable to other approaches or to other national or international regions. All therapists were of a white ethnicity. Participants were self-selecting, though we interviewed a relatively high proportion of young people in each school. The theme-based, qualitative analytical method that we adopted meant that we did not study differences across participants. Our presentation of helpful and hindering factors used a cutoff based on frequency counts, and therefore discounted more minority experiences including those that may have been of greater intensity than the more prevalent ones. In addition, the helpful/hindering binary may occlude nuances and complexities in the data: for instance, aspects of therapy that are initially experienced as hindering but then helpful as the therapy progresses.

Our results have several important implications for practice. First, given the levels of triangulation against previous research, our findings on helpful and hindering therapist qualities and activities provide robust guidance on how humanistic therapists may strive to be, and act, with young people. As Rogers (1957) suggests, being an accepting, caring, and empathic listener is of key importance. However, contrary to a classical “non-directive” stance, there are indications that providing input and guidance—particularly on relational issues and coping strategies—may be of value too. Humanistic therapists should also be mindful of the risks of long silences; maintaining the contact and flow of the session, perhaps even, for instance, using questions, prompts, or creative methods to help young people feel able to communicate and engage in the therapy. Combined with other evidence on the limits of a classical person-centered approach (Elliott et al., 2021), we believe that humanistic therapy for young people may need to support more collaborative direction: with “process guiding,” rather than “non-directivity,” at the heart of the therapeutic work.

Second, given that clients who are informed about—and prepared for—an intervention may be able to make better use of it (e.g., Hoehn-Saric et al., 1964), we believe that young people may benefit from being given clear guidance on what to expect in humanistic therapy. A therapy information sheet, for instance, might list potentially helpful processes of change. This may also help young people decide if the intervention is right or not for them. Equally, prospective clients can be made aware of aspects of the processes that they may find potentially uncomfortable and negative. Sensitizing clients in this way may help them to feel more empowered to raise concerns with their therapists and overcome deference effects (Rennie, 1994).

In terms of future research, the process analysis method we developed has the potential for application in both adolescent and adult psychotherapy research. Our study has demonstrated that such a method of identifying and quantifying processes of change can achieve acceptable levels of inter-rater reliability. By establishing clearly-identifiable cause-and-effect pathways—as perceived by respondents—we believe that it may provide a robust means of identifying mechanisms of change across different therapies, with the potential to compare across client groups (e.g., gender-based or ethnicity-based) and therapeutic approaches. Associations between processes of change and symptom improvement (as assessed, for instance, by nomothetic outcome measures) should also be explored. To support such studies, self-report instruments could also be developed for clients, themselves, to identify processes of change in their therapy. The development of such instruments for parents/carers and other professionals (e.g., teachers) could also then allow comparison across multiple perspectives.

Alongside such process analyses, the development of a more “process-guiding” humanistic therapy for young people could be supported through the use of systematic case studies and repeated longitudinal designs. Furthermore, there is great potential in involving young people themselves in the operationalization of humanistic therapy and in wider service design of school-based interventions. Different methods of co-production can ensure a wider connection between the development of mental health interventions and the lived experience of psychological difficulties. This is particularly poignant with regard to marginalized groups of young people whose perspectives can often be overlooked.

## Supplementary Information

Supplementary Information
