# Supplementary material for: Humanistic Therapy for Young People: Client-Perceived Helpful Aspects, Hindering Aspects, and Processes of Change
Source: J Child Fam Stud. 2025 Jan 14;34(3):686–705. doi: 10.1007/s10826-024-02955-3 (PMC11961482; doi:10.1007/s10826-024-02955-3)
Supplement: Supplementary file 1 — Supplementary Information [file 10826_2024_2955_MOESM1_ESM.docx]

# Humanistic Therapy for Young People: Helpful Aspects, Hindering Aspects, and Processes of Change

# Supplemental Material

# Supplemental Material 1. Interview Topic Guide

**ETHOS: Young People’s Topic Guide**

**Note for researcher: use this topic guide flexibly, it is not a script.**

**Questions are written as a guide and researcher should use and probe as appropriate.**

**Our overall aim is to develop a *rich* and *in-depth* understanding of the participants’ experiences, perceptions and any process of change.**

**Use probes and follow-up questions to help participants ‘unpack’ these as far as they are able and willing to.**

- Introduce self, thank participant for time and speaking to us,
- Go through information sheet and consent form again – reminder of purpose of research, confidentiality and reporting:
  - This research is looking to evaluate the effectiveness of counselling in reducing distress in young people. The key questions we want to answer are:
    - What did you find helpful and unhelpful in School Based Humanistic Counselling?
    - What did you see as any process(es) of change in school based humanistic counselling?
  - This interview is not about issues that you received counselling for
  - There are no right or wrong answers
  - If you want to stop at any point or don’t want to answer a question, then just say so. The important thing is that you feel comfortable
  - What you say to me during this interview will not be shared with teachers, other students, your parents or your counsellor unless you say something that makes me think that you or someone else is at risk of serious harm. I will have to share this with the lead for the project at University of Roehampton.
  - Some of what you say may be used in reports and publications, but nobody will know it is you who said it
- Check permission to record before starting digirecorder:
  - The recording will only be accessed by the research team for the purpose of the research. It will be transcribed confidentially after the interview to allow analysis. The recording and transcript will be stored securely
- Ask if have any questions about what said or the interview
- Check happy to take part and for the interview to start

*Section One: Background Information [keep to five minutes]*

1. Please start by saying a bit about yourself, such as how old you are, where you live, who you live with
   1. How long have you been at school?
   2. What is your favourite subject?
   3. Who’s your favourite teacher?
   4. Tell me about your friends, do they go to this school?

**If feel young person is comfortable, move on to next questions. Remind them that interview is about counselling and any process of change from it.**

1. What did you think when you were offered counselling? (Prompt: did you think it would help you?)
   1. Did you know what counselling was?
   2. What did you expect from the counselling?
   3. What did you want from the counselling?
2. Have you spoken to anyone outside of your family in the past about things that have caused you distress or which you were worried about?

*[If struggling, use following prompts]*

- 1. Who did you speak to?
  2. How did you do this – e.g. did you phone a helpline or chat online to someone, go to see your GP, go to a service for children and young people?
  3. Are you still using any of these services?

1. *Section Two: Your experience of counselling [fifteen minutes]*

I’d like us to draw a model of your experience of counselling please. We have a prepared model here, but you can use a blank piece of paper if you prefer. I can write it for you if you don’t want to [*Allow up to 10 mins for completion]*

1. When was your last session of counselling?
2. Thinking back to all the sessions you had in school with the counsellor, can you talk me through the following and add to the model:
3. Was there anything the counsellor did that you found helpful or unhelpful? [e.g. counsellor seemed friendly; counsellor listened; they seemed to care or take young person’s worries seriously] *Please fill in the* *first column – what counsellor did*
4. How did you respond to this? [e.g. any feelings or actions you took] *Please fill in the second column – how you responded*
5. Any changes, good or bad?[Prompt: this could be changes in your own feelings about yourself, others or your situation; your behaviour or your actions and/or your relationships with others] *Please fill in the third column*
   1. Were these changes helpful or unhelpful? WHY?
6. [For example, why did it make you feel/do this and why was this helpful/unhelpful to you]
7. In the fourth column, please tell me if anything happened as a result of any of these changes [e.g. felt able to join in more at school, able to concentrate/grades improved]
8. *Fill in* *fourth column – what happened next*
9. Looking at your model, I’d like to talk a bit more about whether you see any links between the counselling you received and any changes you’ve identified:
   1. For each change you have mentioned, was there anything specific about the counselling that caused this?
   2. Could anything else have caused these changes? [Prompt: What else was going on in your life at the time, e.g. getting help from somewhere else?]
10. *Section Three: In-depth exploration of process of change [fifteen minutes]*

The following questions should be used flexibly, depending on what has been said in relation to the model.

If feel participant has not mentioned activities listed, then probe.

There has been research on the process of change from school-based counselling before and what people have found helpful or unhelpful. I want to explore your experiences in relation to what this research found.

1. I wanted to ask about some specific things that your counsellor might have done with you, and whether or not you found them helpful or unhelpful. If they were helpful or unhelpful, it would be great if you could say why. So **did or didn’t they**:
   1. **listen carefully** to you? (prompt [if yes or no], Was that helpful/unhelpful? Why?)
   2. **understand** you (prompt [if yes or no], Was that helpful/unhelpful? Why?)
   3. **help** you **express your feelings** (prompt [if yes or no], Was that helpful/unhelpful? Why?)
2. Now I want to ask you some specific things about the kind of person your counsellor might have been with you, whether or not you experienced this, and whether or not it was helpful.
3. Again, if it was helpful or unhelpful, it would be great if you could say why – what kind of effect it had.
4. **Did you or didn’t you**:
   1. feel you could **trust** them (prompt [if yes or no], What was the effect of that?)
   2. feel they were **friendly**
   3. **consistent** (always there, in same place at same time each week, acting in same way each week)
   4. **could you depend** on them
   5. **accepting** of what you had to say
   6. **cared** about you or your views
   7. **independent** (e.g. someone who was separate from the school and your family)
   8. **confidential**
5. Now I’d like to ask you a bit about what YOU did in counselling, and whether or not that was helpful or unhelpful. Remember there are no right or wrong answers. For example, did you or didn’t you talk about how you really felt/what you were experiencing
   1. Was this helpful or not?
6. [Prompts a-e of this question are only to be used if mentioned by the participant at question 9 – probe on relevant helpful things, but if only unhelpful mentioned then go to prompt f] I’d like to ask you about **why** these **things were helpful [e.g. talk/be listened to]** – **how and why did it help you**.
7. [Prompts for if struggling] For example, if you did talk about your genuine feelings, did this help you:
   1. Get things off your chest and feel like you were unburdening yourself of things (prompt: if so, **Why was that useful/how did it help**)
   2. Develop your communication and relationship skills, and feel more able to talk to others outside of counselling about what you’re really feeling (prompt: if so, Why was that useful/how did it help)
   3. Feel more accepting about yourself and what you have been experiencing (prompt: if so, Why was that useful/how did it help)
   4. Understand more about yourself, others, and your situation and why you do things (prompt: if so, Why was that useful/how did it help)
   5. Find ways of doing things that work out better for you (prompt: if so, Why was that useful/how did it help
   6. **If you DIDN’T find it helpful to** [talk about how you were feeling], why do you think this was? How did this make you feel? Could anything have been done differently?
8. Did the counselling give you guidance and advice? Was this in any way helpful or unhelpful?
   1. If you received guidance/advice, how did this make you feel?
   2. Can you talk about whether this had any effect on your ways of coping and stress?
   3. How do you know this was the counselling and not something else?
   4. **If it didn’t** give you advice or guidance, how did this make you feel?
9. Do you think that the counselling had any effect or not on your emotional distress? Can you say why or why not?
   1. If it did change your levels of emotional distress did this change, in any way, your engagement in school? How?
10. Do you think the counselling led to any adverse effects? This means it could have been harmful/negative to you.
    1. [If yes], in what way were you affected by it?
    2. [If yes], how did the counselling do that?
11. [If not discussed] Is there anything you would improve about the counselling?
12. Is there anything else that you want to say about your experience of counselling, the counsellor, or how you feel following the sessions?

Close interview and thank participant.

# Supplemental Material 2. Blank Process Map


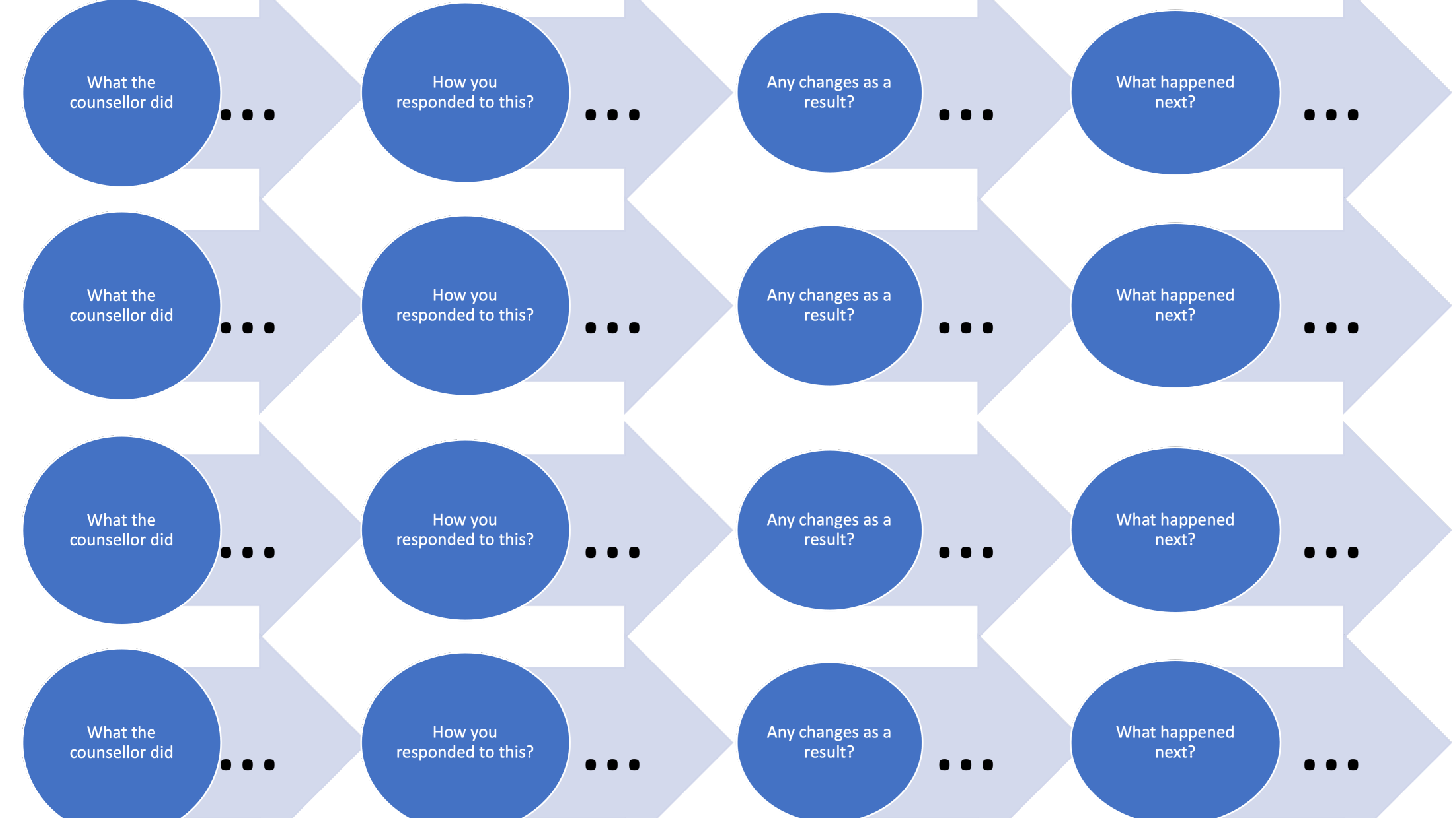


# Supplemental Material 3. Initial Logic Model


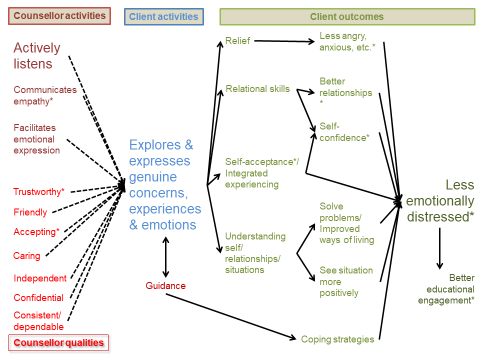


*Source*. [Reference masked for anonymity]

# Supplemental Material 4. Detailed Account of Qualitative Analysis

## Thematic Analysis

The purpose of the thematic analysis (TA) was to identify aspects of the person-centered therapy that the young people had found helpful and hindering. TA is as an umbrella term for a wide variety of qualitative analytical methods that seek to identify, analyze, and report patterns of meaning in data ([Braun & Clarke, 2006](#_ENREF_10), [2022](#_ENREF_11); [Clarke & Braun, 2018](#_ENREF_15)). TA aims to establish *themes*, which capture “something important about the data in relation to the research question” ([Braun & Clarke, 2006, p. 82](#_ENREF_10)). Six phases of the TA process have been proposed: “Familiarizing yourself with your data”, “Generating initial codes”, “Searching for themes”, “Reviewing themes”, “Defining and naming themes”, and “Producing the report” ([Braun & Clarke, 2006](#_ENREF_10)).

Thematic analyses for this study were conducted using NVivo v.11 and v.12. NVivo is a widely-used software program developed for qualitative and mixed-methods research. Coded units of texts were not of a standardized length, and could range from a few words to 2–3 paragraphs (see example of a coded theme in Supplemental Material 5). Text units could be coded into more than one theme. Although client demographic characteristics were stored in NVivo, coding was conducted blind to all such features; except, as in a few cases, when they became evident in the interview transcript.

A preliminary organization of the data, drawing on Braun and Clarke’s ([2006](#_ENREF_10)) six steps of TA, was conducted by Authors 2, 3, and 4. This organized the data into four higher-order themes: “expectations,” “experiences,” “responses,” and “outcomes.” An initial report of this analysis was reviewed by all authors.

The principal analysis was conducted by Authors 1 and 5. This drew on the preliminary organization, but developed domains and subdomains that were more specifically aligned with the research questions being asked. This analysis began with both authors reading through half (*n* = 25) of the interviews each: familiarizing themselves with the data. Following discussions, it was agreed to only code data in which the young people specifically indicated that a factor was helpful or hindering (rather than experiences, per se). It was also agreed to distinguish between responses in the open- and closed-ended parts of the interview. At this stage, Authors 1 and 5 also undertook a self-reflexive exercise—writing down what they, themselves, had found helpful and hindering in their own psychotherapy—as a means of recognizing, and facilitating the bracketing of, assumptions during the analytical process.

The process of thematic analysis progressed iteratively, with interweaving stages of coding, discussion, and reorganization of the analytical frame. Two initial cases were randomly selected, and Authors 1 and 5 open coded (i.e., without any a priori domains or themes) one each. Following discussion and sharing of identified themes, they repeated this process with two further cases. At this point, it was decided that the themes should be organized into several higher-order domains and subdomains, drawn from previous research and scholarship (e.g., Cooper & McLeod, 2011). The domains were (a) *therapist qualities* (i.e., characteristics of the therapist), (b) *therapist activities* (i.e., what the therapist did), (c) *contextual qualities* (i.e., features of the school environment), (d) *client qualities* (i.e., characteristics of the client), (e) *client responses* (i.e., how the client felt in the therapy), (f) *client activities* (i.e., what the client did in the therapy), (g) *immediate outcomes* (in-session, or immediately post-session consequences of the therapy), and (h) *longer-term outcomes* (i.e., what the consequences of the therapy were, beyond the immediate post-session outcomes). The subdomains were what the young people described, within each domain, as *helpful* (i.e., contributing to positive change) and *hindering* (i.e., obstructing positive change).

A further two cases were then randomly selected and coded separately by Authors 1 and 5. These analyses were shared and consensual coding agreed. Themes (and subthemes) were placed within the domain/subdomain structure, and new themes and subthemes were agreed. Author 1 and Author 5 then each analyzed, independently, five more cases; the analyses were merged; and the full analytical frame was then reviewed together for the 16 cases to date. This led to some additional reorganizing, merging, and separation of themes and subthemes. A *Thematic Analysis Codebook* was then produced which gave definitions for each of the elements in the analytical frame, and a further nine interviews were randomly selected and coded by Author 1, with some minor changes to definitions of the themes and subthemes in the *Codebook*. Author 1 and 5 then re-reviewed the analytical frame, and independently coded two further cases, demonstrating inter-coder agreement on 58 out of 74 coding units (78.4%). Author 5 then coded all remaining cases, and the analytical frame was again re-reviewed. Finally, Author 1 went through data in each of the themes and subthemes, recoding small segments of data where appropriate, and then proceeding to write up.

## Process Analysis

The purpose of our process analysis ([Cooper & McLeod, 2015](#_ENREF_19); [McArthur et al., 2016](#_ENREF_37)) was to identify the specific, detectable, pathways of helpful and hindering change in the therapeutic intervention.

This process began with Author 1 creating *process narratives* for each of the 50 interviewees (see example, Supplemental Material 6). These narratives were 2 to 3 paragraph long, and aimed to summarize what each young person concretely described as *processes* *of change* in their therapy. To be coded as a helpful process of change, interview data needed to clearly link particular aspects of the intervention to particular positive, extra-therapeutic outcomes (short- or long-term), forming a coherent and intelligible cause-and-effect chain. For negative process of change, we adopted a more liberal inclusion threshold, given the limited prevalence of such processes. Here, interview data needed to link particular aspects of the intervention to particular negative outcomes, but these could be intra- as well as extra-sessional. An example of the former might be, “When the therapist didn’t listen to me I felt ignored and upset.” Aspects of the therapy that were described as helpful/hindering but had no clearly identified outcomes, or positive/negative outcomes that had no clearly identified causes, were not included in these narratives.

As the development of process narratives progressed, common processes of change (e.g., “getting things off my chest”) were identified and written up, with descriptors, into a *Process analysis codebook* (see Supplemental Material 7). A preliminary coding was then conducted of all 50 interviews by Author 1; with helpful and hindering processes rated, for each young person, as either “Fully Present” (2), “Somewhat Present” (1), or “Not Present” (0).

A second coder (Author 8) was then instructed on the *Process analysis codebook* and asked to code two interviews. Following discussion and feedback, the *Codebook* was refined (with seven helpful processes and six unhelpful processes) and Author 8 coded a further eight interviews. The median Spearman’s correlation at this point between Author 1’s and Author 8’s ratings, across the 13 processes, was ρ = .62.

Following final discussion and clarification of the *Codebook*, two independent Master’s level students then carried out a full coding of all cases (Authors 6 and 7). This consisted of five phases (see Inter-rater reliability, Supplemental Material 8).

The first phase (Phase 1) consisted of training coding with Author 1. As a first step, Authors 6 and 7 read through two randomly selected transcripts independently, identified potential processes of change against the Codebook, and then reviewed their coding with Author 1. Authors 6 and 7 then coded five further transcripts individually and discussed the discrepancies in their coding with Author 1. Pearson’s correlations were checked to see if an agreement of *r* = .7 was achieved between the two coders for each positive and negative processes of change. At this point, there was substantial agreement for *Advice*, *Self-worth*, and *Silences awkward*, but the rest of the processes of change had only slight or fair agreement: from -.17 for *Can’t open up/Trust* to .59 for *Miss lessons*. At this point, it was agreed that processes of change should only be coded as either *Present* (2) or *Not present* (0), with the “1” coding used only for the small number of cases that the coders identified as needing further discussion. There was also agreement to use Cronbach’s Kappa from this point forward to assess inter-coder reliability.

For all subsequent phases, Authors 6 and 7 coded independently of Author 1. Five further randomly selected interviews were coded (Phase 2); but it was found that *Self-worth* and *Creative methods* still did not meet the required level of agreement. Differences in coding were discussed. An additional eight interviews were then coded (Phase 3), with three Kappa values still less than .7. Following further discussion of discrepancies, five further interviews were then coded to ensure inter-rater reliability was established (Phase 4). As this was acceptable (Kappa > .7), the remaining 25 interviews were coded, (Phase 5).

## References

Braun, V., & Clarke, V. (2006). Using thematic analysis in psychology. Qualitative research in psychology, 3(2), 77-101. https://doi.org/10.1191/1478088706qp063oa

Braun, V., & Clarke, V. (2022). Conceptual and design thinking for thematic analysis. Qualitative Psychology, 9(1), 3-26. https://doi.org/10.1037/qup0000196

Clarke, V., & Braun, V. (2018). Using thematic analysis in counselling and psychotherapy research: A critical reflection. Counselling and Psychotherapy Research, 18(2), 107-110. <https://doi.org/10.1002/capr.12165>

Cooper, M., & McLeod, J. (2011). Pluralistic counselling and psychotherapy. Sage.

Cooper, M., & McLeod, J. (2015). Client helpfulness interview studies: A guide to exploring client perceptions of change in counselling and psychotherapy. (Working paper). Retrieved from <https://www.researchgate.net/profile/Mick_Cooper>

McArthur, K., Cooper, M., & Berdondini, L. (2016). Change processes in school-based humanistic counselling. Counselling and Psychotherapy Research, 16(2), 88-99. https://doi.org/10.1002/capr.12061

# Supplemental Material 5. Example of Coded Theme

**Therapist Activities/Hindering/Silences**

**(Coding for first five young people in theme)**

## Young Person 1

### Text Unit 1 (Open-ended section of interview)

Interviewer (I): Is there anything that the counsellor did that you didn’t really like, didn’t think that was helpful?

Young Person (YP): Well, she did do this really awkward thing that made me feel very uncomfortable and not want to come anymore. She’d like– I would speak and I would like, “Yeah.” She goes, “Hmm, yeah. Yeah, hmmm. Yeah.” She just kept on saying, “Hmm, yeah. Hmm, yeah.” I was like, “Hmm.” So it was really awkward, and like the room was silent, and everybody’s sitting there going, “Hmmm.”

I: I’m going to put this down on here as, “Unhelpful,” because I think that’s equally useful, is that okay?

YP: Mm-hm.

## Young Person 2

### Text Unit 1 (Closed-ended section of interview)

I: No, so it was just for that short period of time. Having someone to talk to did help you express yourself to some degree, not fully? Is that fair?

YP: Yes, and most of the time I was going, like, we just sat there in silence because it was, like, I didn’t know what to say or anything, and I don’t think she knew what to say either.

### Text Unit 2 (Closed-ended section of interview)

I: Do you think you would have got more of it if…?

YP: Like if I went silent, she’d go to silent too.

I: How did you find that? Was that helpful or unhelpful?

YP: Unhelpful because it was, like, really off-putting.

I: What could have been done differently?

YP: Well, like, if there was more to talk about and the counsellor was more able to carry on conversation.

## Young Person 3

### Text Unit 1 (Open-ended section of interview)

I: Is there anything conversely that you didn’t like about the sessions? Is there anything she did that you really didn’t like?

YP: It was quiet for most of the time. She didn’t really say how to help me.

I: So it was just quite quiet.

YP: Yes.

I: So how did that make you feel when the sessions were quite quiet? Were there a lot of silences?

YP: Yes.

I: How did that make you feel?

YP: It felt a bit awkward.

### Text Unit 2 (Open-ended section of interview)

I: So we’ve spoken to a lot of other young people across L* and there’s been loads of research on counselling and young people anyway, so yes I think quite a lot of young people said that awkward silences can make them not really want to talk because it’s a bit awkward. It’s a bit, “Oh I don’t really know what to do.” So did you feel like that or did you not really mind the awkward silences?

YP: Yes, I felt a bit like that.

I: Like you didn’t really want to talk, okay. Okay and you said she didn’t really offer too much advice.

YP: Mm.

### Text Unit 3 (Open-ended section of interview)

I: So the point is to make counselling better for other young people that want to go for it, so if you were doing some counsellor training or something, How would you tell the new counsellors to not make it awkward? How would you tell them to avoid these quiet periods?

YP: If it went a bit silent just ask them stuff what they’re doing in school or stuff like what are you doing at the weekend and stuff like that to try and get the silenceness [sic] back up yes, a bit like that.

I: Yes, like small talk kind of thing.

YP: Yes.

I: If you were encouraging your best friend to go to counselling is there anything that you’d tell her about how to fill these awkward silences or what to do to avoid them?

YP: I would just say, “Try and talk as much as you can” and yes just that.

I: So encouraging other young people to talk as much as they want to and encourage counsellors to ask questions and dig a bit and even small talk like, “How’s the weather?” to avoid those awkward silences.

YP: Yes.

### Text Unit 4 (Closed-ended section of interview)

I: Is there anything that you would change about the counselling if another school were to implement it and offer it to their students? Is there anything that you would change based on your experience of it?

YP: I would try and talk more so less quietness, because it was so awkward.

## Young Person 4

### Text Unit 1 (Closed-ended section of interview)

YP: I just didn’t really feel that it was very comfortable for me to talk about my feelings.

I: How do you think they could have made it a bit more comfortable?

YP: Talk more.

I: Talk more, okay, were there quite a lot of silences and stuff when you had your sessions? [Non-verbal response]. Okay, so you think it would have been helpful if they filled those gaps...

YP: Yes.

I: …and spoken a bit more to you? Well, that’s really useful to know, thank you for that.

## Young Person 5

### Text Unit 1 (Open-ended section of interview)

I: On the reverse was there anything about the counsellor that you really didn’t like?

YP: No, it was the silences, just that, yes.

I: How did you feel when there was an awkward silence?

YP: I didn’t know what to say so it made me feel really awkward.

I: Did that impact on your sessions with the counsellor at all?

YP: No, we just had sometimes 20 minutes in silence, but yes.

I: Oh gosh that’s quite a long time.

YP: Yes.

I: Yes, I can imagine that being quite awkward. Was the counsellor asking you things or was it literally 20 minutes in silence?

YP: Just 20 minutes in silence because she didn’t know the questions to ask and stuff.

### Text Unit 2 (Open-ended section of interview)

I: Was that when you started playing games or was that for a different reason?

YP: That’s why we played games yes.

I: You said earlier that you quite liked playing those card games, so I assume playing the games was better than sitting in silence.

YP: Yes.

I: How often did these really awkward silences happen? Was it every session or did they change over the course of the session?

YP: It was every session and we played the games so yes.

I: What else did you do in your sessions? I guess they’re an hour long, so 20 minutes yes quite awkward, a bit of card playing.

YP: We talked about how I was during the week and why I was so up and then played those games.

### Text Unit 3 (Closed-ended section of interview)

I: Can you think of anything that could have been better about the counselling or anything that you would change to make it better?

YP: Just the awkward silences.

# Supplemental Material 6. Thematic Analysis: Domains, Subdomains, and Themes (Extended)

| DOMAIN  *SUBDOMAIN*  Theme | Total (open-ended + closed ended)  *n* participants  (%) | Open-ended only  *n* participants  (%) | Not experienced/not helpful  *n* participants  (%) | Illustrative Extract |
| --- | --- | --- | --- | --- |
| **THERAPIST QUALITIES** |  |  |  |  |
| *HELPFUL* |  |  |  |  |
| **Friendly and welcoming** | **46 (92%)** | **10 (20%)** | **3 (6%)** | **“They didn’t make some formal thing, like a lesson. They were just like, ‘Hi, [name of client’, hi, you’re back,’ and they just welcomed me in.”** |
| **Non-judgmental and unconditionally accepting** | **44 (88%)** | **12 (24%)** | **3 (6%)** | **“I felt like she didn’t judge me, because there was one time I told her about my sexuality, and I said, ‘I’m [sexuality],’ and she goes, ‘Oh, ok.’ I’m like, ‘ok.’”** |
| **Caring** | **37 (74%)** | **10 (20%)** | **8 (16%)** | **“It felt good because it felt like I wasn’t just talking to someone who was just doing their job, it was more like they actually cared.”** |
| **Dependable, reliable, and consistent** | **37 (74%)** | **5 (10%)** | **24 (48%)** | **“She was always there. Like every single session, she was there. She was never late. I’m pretty sure she was early all the time. She was always there and I could always speak to her.”** |
| **Confidential** | **36 (72%)** | **3 (6%)** | **10 (20%)** | **“I can sit there and talk to her about like pretty much anything and I know it’s not going to be said to anyone else for anyone to find out, which makes me feel better.”** |
| **Independent from the school** | **27 (54%)** | **4 (8%)** | **13 (26%)** | **“I found it easier talking to someone who I didn’t really know as well than someone that I knew, which is weird but it’s like I feel like if they know you too well, they’ll maybe hold it against you later on.”** |
| Relaxed, calm, and open | 9 (18%) | 4 (8%) |  | “I think their language, body language, all of that has to do with it. I think it’s just that they were very open, very relaxed and that was very helpful.” |
| A trained, professional, adult | 7 (14%) | 3 (6%) |  | “I knew that she was professional so I knew anything she did say I would at least take on-board and stuff because I knew she was, in a way, kind of like right.” |
| *HINDERING* |  |  |  |  |
| Over-friendly | 5 (10%) | 1 (2%) |  | “She came across a little bit too friendly, and I was like, ‘What's up with her?’ Like, ‘Hmm, she doesn’t come from [local area], that’s for sure.’” |
| **THERAPIST ACTIVITIES** |  |  |  |  |
| *HELPFUL* |  |  |  |  |
| **Listened** | **48 (96%)** | **27 (54%)** | **4 (8%)** | **“I felt like I could say whatever I wanted without her having an opinion straight away. She would listen to everything I had to say.”** |
| **Understanding and empathic** | **43 (86%)** | **14 (28%)** | **8 (16%)** | **“Sometimes she'd put herself in my shoes and saw things from my perspective which was nice and really nice of her because I feel like not a lot of people do that.”** |
| **Advice and guidance** | **39 (78%)** | **24 (48%)** | **18 (36%)** | **“She gave me really good advice on how to deal with situations and to not be upset all the time.”** |
| **Helping the client express feelings** | **24 (48%)** | **3 (6%)** | **12 (24%)** | **“I’d speak a bit something and they’d ask, ‘How do you feel about this? Do you feel distressed? Do you feel upset?’ I did express how I felt with these problems and that helped because then they’d understand how I felt about these problems.”** |
| Creative, artistic, and written media | 19 (38%) | 10 (20%) |  | “If I came in and I had a problem with something and I was a bit angry or something I could do drawing until it calmed me down.” |
| Questions | 18 (36%) | 10 (20%) |  | “He would be like, ‘Well, what are you concerned about and stuff?’ He would challenge my solutions to make sure that I feel like it’s good.” |
| Not forcing or rushing the young person to talk | 16 (32%) | 6 (12%) |  | “They gave me the space, like they didn’t constantly ask me questions and they just gave me a bit of space to think, time.” |
| Reflecting and rephrasing | 10 (20%) | 5 (10%) |  | “Quite often when talking about things like that was going on she would wait for me to finish before starting talking and then if she didn’t understand she would say, ‘You’ve said this and this is what I get out of it, is this right or do you mean something else?’” |
| Insights and new perspectives | 6 (12%) | 4 (8%) |  | “When she gave her thoughts, it kind of made me see things differently as well, so say I’m upset they would be- we’ll speak about why I’m upset, and she’ll try and see it in a different perspective. Maybe I’m overthinking it or something.” |
| Remembering | 11 (22%) | 2 (4%) |  | “They might refer back to something and it was just like linking all those things, it was quite helpful.” |
| Self-disclosure | 6 (12%) | 1 (2%) |  | “She is open about herself as well, which gave me more insight, and like, ‘well, she’s opened up to me, now I have to open up to her.’” |
| *HINDERING* |  |  |  |  |
| Silences | 13 (26%) | 9 (18%) |  | “She did do this really awkward thing that made me feel very uncomfortable and not want to come anymore. She’d like- I would speak and I would like, ‘Yeah.’ She goes, ‘Hmm, yeah. Yeah, hmm. Yeah.’ She just kept on saying, ‘Hmm, yeah. Hmm, yeah.’ I was like, ‘Hmm.’ So it was really awkward, and like the room was silent, and everybody’s sitting there going, ‘Hmm.’” |
| Insufficient activity: advice, guidance, strategies, activities, questions | 12 (24%) | 5 (10%) |  | “I would just, like- it if the counselor gave a bit more advice or suggestions because sometimes I would just speak and then she wouldn’t give any advice or anything and then I’d feel lost.” |
| Guidance, activities | 6 (12%) | 4 (8%) |  | “[The therapist] would do breathing exercises and imagine things, and I didn’t like that. I said, ‘I didn’t like that,’ and [the therapist] was like, ‘Well, let's just try.’ ‘I don’t want to do it.’ ‘Well, let’s just try.’ [The therapist] was kind of persistent on something that I didn’t want to do.” |
| **CONTEXTUAL QUALITIES** |  |  |  |  |
| *HELPFUL* |  |  |  |  |
| Consistent time, place | 7 (14%) | 0 (0%) |  | “It was the same day, same place and I guess the same place made it easier because I knew the environment.” |
| *HINDERING* |  |  |  |  |
| Missed classes | 11 (22%) | 2 (4%) |  | “I stopped going [to the therapy] because I was missing out on the key lessons that I had to go to, like the ones that I was struggling in and then I would just go back into the lesson and I wouldn’t understand what they were doing.” |
| Not enough sessions | 5 (10%) | 0 (0%) |  | “If people did counseling more than once a week that might help, yes.” |
| **CLIENT QUALITIES** |  |  |  |  |
| *HINDERING* |  |  |  |  |
| Dislike talking, shyness | 8 (16%) | 6 (12%) |  | “It’s just because I'm such a shy person, I’m quite shy, so I think that just helped as well [stopping the therapy], because it was like talking about myself for an hour. I just didn’t like it.” |
| **CLIENT RESPONSES** |  |  |  |  |
| *HELPFUL* |  |  |  |  |
| **Felt trust** | **40 (80%)** | **4 (8%)** | **8 (16%)** | **“I feel like the stuff I told her, that she didn’t tell anyone, that I could trust her with the things I was saying. If I was putting myself in danger then, yes, she can tell other people.”** |
| Felt free to talk and open up | 35 (70%) | 18 (36%) |  | “The stuff that I didn’t feel comfortable saying to my parents I felt comfortable saying in the room. I particularly felt I could get everything out, because I kept everything in, so it was nice to keep it out because they kept it private so it’s nice that I get to say it out loud.” |
| Felt comfortable, relaxed, and not judged | 28 (56%) | 16 (32%) |  | “We were on a level where we were kind and happy with the way that we were talking, and it wasn’t just, ‘Oh, I have to speak about what's going on… Great.’ It was like, ‘Oh, here’s my counsellor. I can talk to her about what’s going on today or what’s happened in the past week.” |
| Felt happy, supported, and cared for | 22 (44%) | 17 (34%) |  | “It made me feel- I don’t know. It made me feel just like I had someone behind me.” |
| Greater self-reflection, different perspectives | 18 (36%) | 11 (22%) |  | “[The therapy gave] me time to think how I was feeling and making- giving me time to realize, really, because most of the time I just get put in a situation. I never really have time to think, ‘Oh how am I feeling right now? What’s going on?’” |
| Felt understood | 11 (22%) | 3 (6%) |  | “It felt as if she knew me. Like, not ‘knew me’ but she could understand me.” |
| *HINDERING* |  |  |  |  |
| Felt awkward, uncomfortable, or weird | 13 (26%) | 9 (18%) |  | “Sometimes there’d be points where, after I’d said something, [the therapist] would just stare at me, and there would be an awkward silence for ten seconds and [the therapist would] be like, ‘Oh…’ Then, I’d be like, ‘I don’t really know what else to say.’ [The therapist] would be like, ‘Do you just want to finish now?’ or something, so it was a bit awkward at times.” |
| Felt like they wanted to drop out or did drop out | 10 (20%) | 9 (18%) |  | “I was always like paranoid like, ‘Oh, I'm boring her.’ To be honest, there were times when I just wanted to walk out and just walk home, I hate it and, you know, yes- there were times I just didn’t know what to say and I just had to sit there, like ‘I don't know what to say.’” |
| Felt they had not had enough to talk about | 5 (10%) | 3 (6%) |  | “I just used to sit there and rant, but there was just that awkward stuff that sometimes I was just like, ‘I don't know what to tell you because everything is the same, nothing’s changed in my still depressing kind of life, so I don’t know what to like really tell.” |
| **CLIENT ACTIVITIES** |  |  |  |  |
| *HELPFUL* |  |  |  |  |
| **Express feelings, get things off their chest** | **47 (94%)** | **26 (52%)** | **5 (10%)** | **“You’ve got all the negativity inside of you and you’ve got the positive inside of you but you’re drawing all the negativity out because you’re being able to express the negativity to someone else.”** |
| Took advice | 14 (28%) | 10 (20%) |  | “I got [useful guidance] from the sessions because if I said to the counselor, ‘This happened,’ then she might’ve been like, ‘Well, maybe next time try this. Don't be so like that, be like that.’” |
| Putting in effort, planning, “giving it a go” | 11 (22%) | 2 (4%) |  | “I tried to open up to her as much as possible…. I’d be going way deep in stuff, which I haven’t really done before. I don’t know, I'm just getting my confidence. I was just trying to be confident with her and just keep telling myself, you know, what- ‘It’s fine, she’s here to help me and you might help me, why not consider it?’” |
| Find their own answers | 7 (14%) | 3 (6%) |  | Interviewer: “Did the counselor give you guidance or advice at all on anything in terms of how to deal with those situations?” Young Person: “No, she just made me speak my own advice. She didn’t tell me how to- like she didn’t advise me anything. She just made me realize things for myself.” |
| *HINDERING* |  |  |  |  |
| Didn’t open up | 7 (14%) | 4 (8%) |  | “I just hated it. I don’t know why. I like- there’s only one reason I know why, it’s because I don’t like showing my feelings.” |
| **IMMEDIATE OUTCOMES** |  |  |  |  |
| *HELPFUL* |  |  |  |  |
| **Greater insight and self-understanding** | **34 (68%)** | **8 (16%)** | **6 (12%)** | **I spoke about certain situations where it [anger triggered] happened and then we realized that certain things make me feel this way and how certain things can help dimmer [sic] the anger.’** |
| **Unburdened, relieved, a “weight” lifted** | **32 (64%)** | **14 (28%)** | **5 (10%)** | **“Once I’d get something off my chest, I’d go back to lessons and I wouldn’t feel so heavy weighted on subjects that didn’t really apply to school. My school work would be so much easier for me to look at and go, ‘Yes, I can do that,’ rather than thinking about things that I didn’t need to think about.”** |
| Calmer, less anxious, and more positive | 23 (46%) | 18 (36%) |  | “It was quite calming. I had less stress and anxiety because just after we spoke, it was calm and quiet.” |
| Feeling there was someone to talk to | 20 (40%) | 9 (18%) |  | “It made me, in general, a happier person because I knew that I wouldn’t have to go around thinking- worrying about everybody else. I could have a time where I could just have time where I could think about what was going on in my life, rather than revolving it around others.” |
| *HINDERING* |  |  |  |  |
| More negative feelings and behaviors | 6 (12%) | 5 (10%) |  | “It felt a bit worse, but not because of anything that she said. It’s just when you talk about all the bad stuff, it just makes you feel a bit bad, as well.” |
| **LONGER-TERM OUTCOMES** |  |  |  |  |
| *HELPFUL* |  |  |  |  |
| **Improvement in relationships** | **42 (84%)** | **24 (48%)** | **24 (48%)** | **“At the start, I didn’t really trust a lot of people outside of counseling and then I started to have a different perspective on other people, so obviously I had a lot more trust built up.”** |
| **Reductions in emotional distress** | **35 (70%)** | **23 (46%)** | **7 (14%)** | **“It made me, in general, a happier person. Even still, now, I have more of a positive mindset than I used to.”** |
| **Improvements at school** | **34 (68%)** | **16 (32%)** | **16 (32%)** | **“I just can carry on with my day and focus on my schoolwork and just not focus on anything else. Like I’m just focusing on my work where before I wouldn’t really focus on it. Like I wouldn’t actually care about what I did in my class where now I do.”** |
| **Improved coping strategies, resilience, and self-control** | **29 (58%)** | **11 (22%)** | **5 (10%)** | **“I feel much more positive, just in general…I can just take some time to slow down and use the strategies that we talked about, yes, keep using the things that we talked about during the counseling sessions.”** |
| **Increased self-acceptance** | **27 (54%)** | **3 (6%)** | **10 (20%)** | **“It made me feel a lot more positive about myself. Like, before I started counseling I didn’t like anything about myself, I thought I was just useless. And now I would say, ‘Yes, I’m not perfect, but I can improve it by doing this,’ or, ‘I’m the best I can be with this and that.’”** |
| Improved confidence, and self-esteem | 19 (38%) | 5 (10%) |  | “It made me feel more confident about me being in the society, kind of, like being myself. Yes, be myself, not worrying about what people think about me.” |

Note. **Emboldened** **themes** were specifically enquired into in the closed-ended part of the interview. Cutpoint for inclusion: Helpful aspects ≥ 10% of young people, hindering aspects ≥ 10% of young people, across both parts of the interview. Some extracts have been further anonymized to ensure participant confidentiality.

# Supplemental Material 7. Example of Process Narrative

YP didn’t expect counselling to be great, and initially didn’t want to do it. She said that she’s not very good with trust and didn’t really feel she could depend on T or felt that T cared about her. YP said, “I just don’t like people a lot.” Drawing with felt tips and coloring pens through the sessions helped YP to feel less anxious. That helped her feel “a bit less nervous around people.” YP did get some advice from T and said that was helpful and “block out everything else.” YP also said that it was helpful that she could talk about anything. YP felt a bit listened to and safe due to the confidentiality, but it didn’t change anything. YP said that she, “didn’t really feel that it was very comfortable for me to talk about my feelings.” She would have liked T to talk more. YP felt like people were staring at her on the way to sessions and that made her feel a bit more stressed.

*Note*. YP = young people, T = therapist.

# Supplemental Material 8. Process Analysis Codebook (Final version)

**Helpful/Unhelpful Processes of Change**

**Processes of Change**

- *Processes of change* are pathways by which specific elements of an intervention are described as leading to specific outcomes.
- Process of change are not simply in-counselling experiences, per se, but experiences that are described by clients as *leading to* particular consequences: that is, where there is *a coherent and clear intelligible pathway* between the counselling element and the outcome. For example, a client describing a counsellor as ‘kind’ would not, in itself, be a pathway of change. However, if the client said that the counsellor’s kindness led them to be more kind to themselves, then that would be the kind of pathway we are aiming to identify.
- If a client describes an element of counselling that is the starting point of a pathway (for instance, getting advice), but then says that there was no helpful outcome to it, do not code as a helpful pathway of change.
- Pathways need to be things that clients *actually* *experienced* in the intervention, rather than things that they expected, wanted, or imagined would happen.
- For the present coding, we are only focusing on what the ETHOS counsellor did. If the client is referring to change in relation to another professional or adult, do not code.
- Please bear in mind the ‘demand characteristics’ of the interview exchange, particularly the second structured part, meaning that clients may experience some obligation to agree with the interviewer or respond ‘yes’ to their questions. Hence, more weight should be put on answers that are given spontaneously, without prompting (particularly in the first, unstructured part of the interview, see below). If a client simply responds ‘yes’ to a process but does not elaborate it in any way, code as ‘somewhat present’ rather than ‘fully present.’
- Note: the unhelpful pathways of change tend to be shorter term than the helpful pathways, describing either immediate negative outcomes or impediments to positive therapeutic change.

**The Interviews**

- The interviews are with clients after up to 10 weeks of counselling.
- The clients are of mixed genders and ethnicities, from ages 13 -16 (details are not giving to minimise any biases in the coding).
- The interviews generally took place within a month after counselling was completed.
- In the first part of the interview, the client was asked to describe, in their own words, processes of change that took place for them in counselling. To support this process they were asked to fill in a ‘process diagram’. In the second part of the interview, the client was asked a series of structured questions, based on previous understandings of processes of change in school-based counselling.
- The process diagrams should be consulted as they may help you to understand any pathways of change being described by the client. However, they are not always describing specific pathways of change (as defined, above), and may not be easily interpretable. They should be used as an auxiliary tool for the coding, therefore, rather than the principal basis for your assessment.

**Instructions**

- Please carefully read through each interview and code them on the coding sheet according to whether each of the helpful and unhelpful change processes were present or not (2 = ‘Present’, 1 = ‘Possibly present,’ 0 = ‘Not present’).
- Keep a record of the segments of text that you have coded as pathways of change by using the ‘comments’ function on Word, or by highlighting in different colours.
- Your final coding should be based on the interview as a whole. For instance, if a client describes a helpful pathway, but later retracts that or explains that, actually, that did not take place, then do not code as a pathway of change.
- There may be many sections of the interview where processes of change are not described, and it is quite legitimate to leave large tracts uncoded. Please bear in mind that there have been other analyses about particular helpful and unhelpful factors (e.g., kindness, concentration at school), and this analysis is specifically focused on *processes* that stretch from intervention to outcomes.
- For each interview, you can code as many change processes as is appropriate.
- If you identify any processes of change, helpful or unhelpful, that are not identified in the grid, please note then down along with the client number.

**Rating of Processes**

- *‘Present’ (2):*
  - Process is explicitly and spontaneously stated in interview, particularly in open-ended (first) part of interview or in process diagram.
  - Links clearly made by client to helpful/unhelpful outcome.
  - Process is described more than once (or one mention but clearly spontaneous and/or very strongly emphasised).
  - Client describes longer term effects of process: e.g., affecting their relationships or their wellbeing outside of counselling.
  - May be only one mention of process.
  - Process is implicit in interview but good evidence that it has been experienced by the client.
- *‘Not present’ (0)*
  - Process is not discussed in interview.
  - May emerge only through prompted.
  - Elements of process of may be mentioned, briefly, but no links (explicit or implicit) to helpful/unhelpful outcomes
  - Positive response is given but just as very brief answer to interviewer’s questions, and likely to indicate compliance rather than a genuine experiencing of the pathway.
  - Different elements are described but may not be clearly linked together.

**Overall Ratings of Helpfulness**

- In addition to rating the helpful and unhelpful processes, we would like you to give an overall rating of how helpful the counselling seems to have been for the young person. This rating should be a number between 1 and 10:
  - 1 = Not at all helpful
  - 10 = Extremely helpful

**Categories of Responses**

- Finally, related to the ratings above, we would like you to categorise each client into one of four groups:
  - *Pos* = Entirely, or almost entirely, positive about the counselling experience.
  - *Pos–* = Generally positive about the counselling experience, but with one or two areas where they are more negative.
  - *Neg+* = Generally negative or nonplussed about the counselling experience (i.e., it wasn’t particularly helpful), but with one or two elements that they describe as more helpful.
  - *Neg* = Wholly or almost entirely negative about the counselling experience.

| **Title** | **Definition** |
| --- | --- |
| HELPFUL |  |
| **Modelling Relationships (Modelling)** | YP reported that the experiencing of a positive relationship with therapist (T) translated into more positive relationships with others in their lives. For example, YP described being more trusting of people in their lives (such as family or friends), or being more open with them, because they had experienced trust towards T and been able to open out to T. Example quote: 'If I can trust someone I don't even know, and tell them things [i.e., T], I think I can trust my friends.' Finding it helpful to ‘role play’ relationships with T should be coded here, but direct guidance from T to YP on interpersonal relating and skills should not be coded here but under Advice and Guidance. |
| **Getting Things off their Chest (Off chest)** | YP reported that, in the counselling, they could ‘offload’/‘get their feelings, emotions, and problems out’; which led to unburdening and relief: reduced stress, reduced anger, and/or greater ability to focus, concentrate, ‘clearing head’, and feelings of calm. For instance, ‘It was getting all the negativity out of me'; ‘Where I speak about everything, it's all gone and then like I can carry on with my day without having to think about it, which makes me feel better about it.' |
| **Advice and Guidance (Advice)** | YP reported that T offered them suggestions and input—for instance ‘guidance’, ‘advice’, ‘opinions’, ‘options’, or ‘alternatives’—that YP experienced as helpful: things that they could apply in their life to positive benefit. This includes T suggesting coping strategies; ways of dealing positively with interpersonal and/or school situations; reassurance; and resources, such as books and websites. |
| **Insight to Behaviour Change (Insight)** | Through therapy, YP reported developed a greater understanding/awareness of their feelings, thoughts, experiences, and problems—and/or the behaviour or perspectives of others—which then helped them find more positive ways of being and behaving. Code the development of different perceptions of their problems as insight (rather than advice). Self insights, per se, shouldn’t be coded here unless clear evidence that that then led to change. |
| **Developing Self-Worth (Self-worth)** | YP describes coming to feel better about themselves (greater self-worth, self-esteem) as a consequence of one or more processes: e.g., being accepted by T, being listened to by T, positive feedback/caring from T, having their self-criticism challenged. For instance, YP recognising that they’re, 'not worthless. I am a human and I have got feelings like everybody else'. |
| **Awareness of Support (Support)** | YP reported that, knowing T was there to talk to and that they would be seeing T (for instance, the following week), made them feel less stressed and anxious during the week. |
| **Learning Creative Methods (Creative)** | YP described T introducing ‘creative’ or play methods—like drawing, cards, or the use of a ‘stress ball’—that T could apply helpfully in the external world (e.g., as a means of stress reduction). Note, should only be coded as present if led to application of creative method in outside world, not if creative methods experienced as positive, per se. |

| **UNHELPFUL** |  |  |
| --- | --- | --- |
| **Silences Awkward (Silence)** | YP reported silences as unhelpful: for instance saying that they felt awkward or uncomfortable during them, or that they led to YP dropping out/wanting to dropout. For instance, ‘She would just sit there and stare at me for sometimes three minutes at a time. Literally it was three minutes. She'd just sit there, and I'd be looking around the room, and every time I looked back at her, she's just looking at me.’ |  |
| **More Input Wanted (Input)** | YP described wanting more input from T: for instance, advice, strategies, feedback, or for T to talk more. |  |
| **Can’t Open Up/Trust (No trust)** | YP described finding counselling difficult because they found it hard to trust T/open up to T: for instance, YP describes themselves as a ‘shy’ person and/or someone who finds it hard to talk about their feelings (*and* therefore found it hard to talk to T, not just shy per se). If client indicates some difficulties trusting at first, but then goes on to develop trust, code as ‘1’. |  |
| **Unnatural, Clichéd (Unnatural)** | YP described it as unhelpful that T, or the therapeutic process, was not genuine or natural: for instance, ‘clichéd’, ‘cheesy’, ‘inauthentic’, ‘weird’; or that T was ‘too nice’ or ‘robotic’. |  |
| **Feel Worse** | YP reported coming out of the sessions, in some respects, feeling worse: for instance, more angry, or upset |  |
| **Miss Lessons** | YP said that missing classes/lessons because of therapy, or missing work, had some form of negative impact. |  |

**Coding Table**

2 = ‘Present’

0 = ‘Not present’

| Participant code | Model-ing | Off chest | Advice | Insight | Self-worth | Support | Creative | Silence | Input | Can’t open | Un-natural | Feel worse | Miss lessons | Overall Rating of Helpfulness | Category  Pos  Pos-  Neg+  Neg |
| --- | --- | --- | --- | --- | --- | --- | --- | --- | --- | --- | --- | --- | --- | --- | --- |
|  |  |  |  |  |  |  |  |  |  |  |  |  |  |  |  |
|  |  |  |  |  |  |  |  |  |  |  |  |  |  |  |  |
|  |  |  |  |  |  |  |  |  |  |  |  |  |  |  |  |
|  |  |  |  |  |  |  |  |  |  |  |  |  |  |  |  |
|  |  |  |  |  |  |  |  |  |  |  |  |  |  |  |  |
|  |  |  |  |  |  |  |  |  |  |  |  |  |  |  |  |
|  |  |  |  |  |  |  |  |  |  |  |  |  |  |  |  |
|  |  |  |  |  |  |  |  |  |  |  |  |  |  |  |  |
|  |  |  |  |  |  |  |  |  |  |  |  |  |  |  |  |
|  |  |  |  |  |  |  |  |  |  |  |  |  |  |  |  |
|  |  |  |  |  |  |  |  |  |  |  |  |  |  |  |  |
|  |  |  |  |  |  |  |  |  |  |  |  |  |  |  |  |
|  |  |  |  |  |  |  |  |  |  |  |  |  |  |  |  |
|  |  |  |  |  |  |  |  |  |  |  |  |  |  |  |  |
|  |  |  |  |  |  |  |  |  |  |  |  |  |  |  |  |
|  |  |  |  |  |  |  |  |  |  |  |  |  |  |  |  |
|  |  |  |  |  |  |  |  |  |  |  |  |  |  |  |  |
|  |  |  |  |  |  |  |  |  |  |  |  |  |  |  |  |
|  |  |  |  |  |  |  |  |  |  |  |  |  |  |  |  |
|  |  |  |  |  |  |  |  |  |  |  |  |  |  |  |  |
|  |  |  |  |  |  |  |  |  |  |  |  |  |  |  |  |
|  |  |  |  |  |  |  |  |  |  |  |  |  |  |  |  |
|  |  |  |  |  |  |  |  |  |  |  |  |  |  |  |  |

Table continued)

|  | Model-ling | Off chest | Advice | Insight | Self-worth | Support | Creative | Silence | Input | Can’t open | Un-natural | Feel worse | Miss lessons | Overall Rating of Helpfulness | Category  Pos  Pos-  Neg+  Neg |
| --- | --- | --- | --- | --- | --- | --- | --- | --- | --- | --- | --- | --- | --- | --- | --- |
|  |  |  |  |  |  |  |  |  |  |  |  |  |  |  |  |
|  |  |  |  |  |  |  |  |  |  |  |  |  |  |  |  |
|  |  |  |  |  |  |  |  |  |  |  |  |  |  |  |  |
|  |  |  |  |  |  |  |  |  |  |  |  |  |  |  |  |
|  |  |  |  |  |  |  |  |  |  |  |  |  |  |  |  |
|  |  |  |  |  |  |  |  |  |  |  |  |  |  |  |  |
|  |  |  |  |  |  |  |  |  |  |  |  |  |  |  |  |
|  |  |  |  |  |  |  |  |  |  |  |  |  |  |  |  |
|  |  |  |  |  |  |  |  |  |  |  |  |  |  |  |  |
|  |  |  |  |  |  |  |  |  |  |  |  |  |  |  |  |
|  |  |  |  |  |  |  |  |  |  |  |  |  |  |  |  |
|  |  |  |  |  |  |  |  |  |  |  |  |  |  |  |  |
|  |  |  |  |  |  |  |  |  |  |  |  |  |  |  |  |
|  |  |  |  |  |  |  |  |  |  |  |  |  |  |  |  |
|  |  |  |  |  |  |  |  |  |  |  |  |  |  |  |  |
|  |  |  |  |  |  |  |  |  |  |  |  |  |  |  |  |
|  |  |  |  |  |  |  |  |  |  |  |  |  |  |  |  |
|  |  |  |  |  |  |  |  |  |  |  |  |  |  |  |  |
|  |  |  |  |  |  |  |  |  |  |  |  |  |  |  |  |
|  |  |  |  |  |  |  |  |  |  |  |  |  |  |  |  |
|  |  |  |  |  |  |  |  |  |  |  |  |  |  |  |  |
|  |  |  |  |  |  |  |  |  |  |  |  |  |  |  |  |
|  |  |  |  |  |  |  |  |  |  |  |  |  |  |  |  |
|  |  |  |  |  |  |  |  |  |  |  |  |  |  |  |  |
|  |  |  |  |  |  |  |  |  |  |  |  |  |  |  |  |
|  |  |  |  |  |  |  |  |  |  |  |  |  |  |  |  |
|  |  |  |  |  |  |  |  |  |  |  |  |  |  |  |  |

# Supplemental Material 9. Inter-Rater Reliability on Processes of Change

|  | **Phase 1** | **Phase 2** | **Phase 3** | **Phase 4** | **Phase 5** | **Overall** |
| --- | --- | --- | --- | --- | --- | --- |
|  | **(*n* = 7)** | **(*n* = 5)** | **(*n* = 8)** | **(*n* = 5)** | **(*n* = 25)** | **(*N* = 50)** |
| Modelling relationships | .36 | 1.00 | 1.00 | .62 | .91 | .84 |
| Getting things off their chest | .30 | 1.00 | .50 | 1.00 | .75 | .69 |
| Advice and guidance | 1.00 | 1.00 | .60 | 1.00 | .90 | .91 |
| Insight to behavior change | .36 | 1.00 | 1.00 | 1.00 | .84 | .84 |
| Developing self-worth | .72 | -.25 | 1.00 | 1.00 | .92 | .84 |
| Awareness of support | NA | 1.00 | 1.00 | NA | .88 | .88 |
| Learning creative methods | NA | .55 | 1.00 | NA | .88 | .79 |
|  |  |  |  |  |  |  |
| Silences awkward | 1.00 | 1.00 | 1.00 | 1.00 | .90 | .95 |
| More input wanted | .30 | 1.00 | .38 | 1.00 | 1.00 | .81 |
| Can’t open up/trust | -.17 | 1.00 | .71 | NA | .88 | .72 |
| Unnatural/clichéd | NA | 1.00 | NA | .55 | 1.00 | .93 |
| Feel Worse | NA | 1.00 | 1.00 | 1.00 | .65 | .83 |
| Miss Lessons | .59 | 1.00 | 1.00 | NA | .78 | .83 |

*Note*. NA = No young people coded within this process of change.

# Supplemental Material 10. Point Biserial Correlations between Processes of Change and Demographic Characteristics

|  |  | 1 | 2 | 3 | 4 | 5 | 6 | 7 | 8 | 9 | 10 | 11 | 12 | 13 | 14 | 15 | 16 |
| --- | --- | --- | --- | --- | --- | --- | --- | --- | --- | --- | --- | --- | --- | --- | --- | --- | --- |
| 1 | Modelling |  |  |  |  |  |  |  |  |  |  |  |  |  |  |  |  |
| 2 | Off chest | .30* |  |  |  |  |  |  |  |  |  |  |  |  |  |  |  |
| 3 | Advice | .21 | .25 |  |  |  |  |  |  |  |  |  |  |  |  |  |  |
| 4 | Insight | .28 | .25 | .24 |  |  |  |  |  |  |  |  |  |  |  |  |  |
| 5 | Self-worth | .37* | .39* | .27 | .44* |  |  |  |  |  |  |  |  |  |  |  |  |
| 6 | Support | -.11 | .09 | -.13 | .22 | .07 |  |  |  |  |  |  |  |  |  |  |  |
| 7 | Creative | .04 | -.05 | -.17 | -.02 | .12 | -0 |  |  |  |  |  |  |  |  |  |  |
| 8 | Silence | -.17 | -.14 | -.02 | -.11 | -.24 | .1 | -.2 |  |  |  |  |  |  |  |  |  |
| 9 | Input | -.21 | -.01 | -.36* | -.07 | -.02 | .07 | .11 | .27 |  |  |  |  |  |  |  |  |
| 10 | No trust | -.21 | -.69* | -.25 | -.43* | -.48* | -.1 | .05 | .24 | .11 |  |  |  |  |  |  |  |
| 11 | Unnatural | -.16 | -.36* | -.26 | -.24 | -.2 | .03 | .05 | .34* | .31* | .36* |  |  |  |  |  |  |
| 12 | Feel Worse | -.11 | -.29* | -.20 | -.07 | -.27 | .06 | .09 | .13 | -.00 | .29* | .45* |  |  |  |  |  |
| 13 | Miss Lessons | .13 | -.02 | .05 | -.19 | -.27 | -.1 | -.2 | .01 | -.14 | -0.16 | -.02 | 0 |  |  |  |  |
| 14 | Age | **.10** | **.19** | **.29*** | **-.20** | **-.03** | **-.17** | **-.24** | **.18** | **.09** | **.02** | **-.04** | **-.18** | **.02** |  |  |  |
| 15 | Gender | **-.17** | **-.34*** | **-.13** | **-.14** | **-.11** | **-.05** | **.12** | **-.09** | **-.11** | **.20** | **.01** | **-.03** | **-.15** | -.14 |  |  |
| 16 | Ethnicity | **-.10** | **-.07** | **-.02** | **.05** | **.03** | **.06** | **.31*** | **-.06** | **.09** | **.07** | **-.13** | **.26** | **-.09** | -.21 | .08 |  |
| 17 | Disability | **-.25** | **-.26** | **-.05** | **-.22** | **-.06** | **-.18** | **.00** | **.09** | **.07** | **.10** | **.22** | **.06** | **.06** | .06 | .08 | -.01 |

*Note.* *N* = 50, *p < .05. Female = 0, male & other = 1; White ethnicity = 0, Black, mixed, other = 1; 0 = no disability, 1 = disability. **Emboldened** correlations are those between the processes of change and the young people’s demographic characteristics.
